# Structure-Activity Relationship Modeling and Experimental Validation of the Imidazolium and Pyridinium Based Ionic Liquids as Potential Antibacterials of MDR *Acinetobacter baumannii* and *Staphylococcus aureus*

**DOI:** 10.3390/ijms22020563

**Published:** 2021-01-08

**Authors:** Ivan V. Semenyuta, Maria M. Trush, Vasyl V. Kovalishyn, Sergiy P. Rogalsky, Diana M. Hodyna, Pavel Karpov, Zhonghua Xia, Igor V. Tetko, Larisa O. Metelytsia

**Affiliations:** 1 V.P. Kukhar Institute of Bioorganic Chemistry and Petrochemistry, National Academy of Science of Ukraine, 1 Murmanska Street, 02660 Kyiv, Ukraine; ivan@bpci.kiev.ua (I.V.S.); maria@bpci.kiev.ua (M.M.T.); vkovalishyn@bpci.kiev.ua (V.V.K.); sergey.rogalsky@gmail.com (S.P.R.); hodyna@bpci.kiev.ua (D.M.H.); metelitsa@bpci.kiev.ua (L.O.M.); 2 Institute of Structural Biology, Helmholtz Zentrum München—German Research Center for Environmental Health (GmbH), Ingolstädter Landstraße 1, D-85764 Neuherberg, Germany; pavel.karpov@helmholtz-muenchen.de (P.K.); zhonghua.xia@helmholtz-muenchen.de (Z.X.); 3 BIGCHEM GmbH, Unterschleißheim, Valerystr. 49, D-85716 Neuherberg, Germany

**Keywords:** ionic liquids (ILs), *Acinetobacter baumannii*, *Staphylococcus aureus*, enoyl-ACP reductase, FabI, OCHEM, imidazolium, pyridinium, guanidinium, Structure-Activity modeling, QSAR

## Abstract

Online Chemical Modeling Environment (OCHEM) was used for QSAR analysis of a set of ionic liquids (ILs) tested against multi-drug resistant (MDR) clinical isolate *Acinetobacter baumannii* and *Staphylococcus aureus* strains. The predictive accuracy of regression models has coefficient of determination q^2^ = 0.66 − 0.79 with cross-validation and independent test sets. The models were used to screen a virtual chemical library of ILs, which was designed with targeted activity against MDR *Acinetobacter baumannii* and *Staphylococcus aureus* strains. Seven most promising ILs were selected, synthesized, and tested. Three ILs showed high activity against both these MDR clinical isolates.

## 1. Introduction

**Figure 1:**
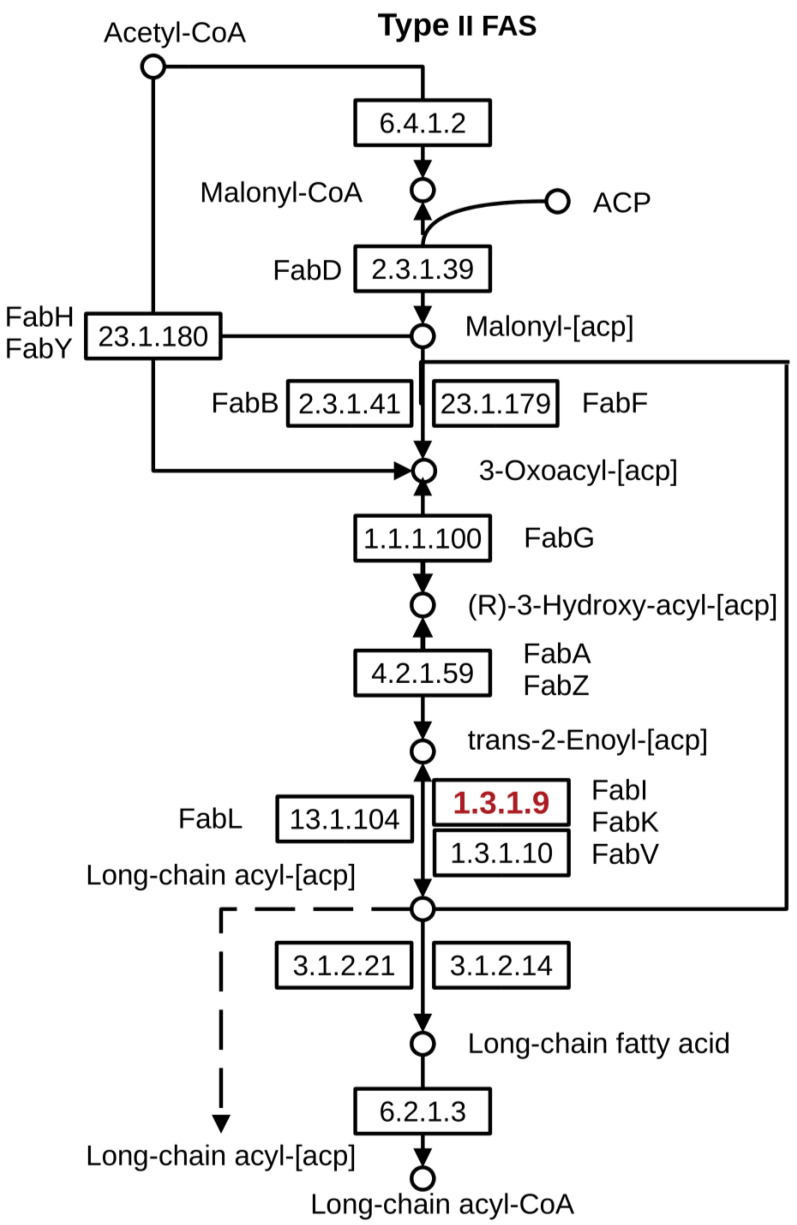
Key role of enzyme FabI (highlighted in red) in *A. baumannii* and *S. aureus* fatty acid biosynthesis [15].

The World Health Organization has included the bacterial pathogens *Acinetobacter baumannii* and *Staphylococcus aureus* with high levels of antibiotic resistance in the list of “priority microorganisms” that pose the greatest threat to human health and require urgent action to develop new antimicrobial agents [1]. These bacteria are found in hospitals and are very effective human colonizers [2,3]. The research and development of new antibacterials with the purpose of overcoming microbial multidrug resistance to known antibiotics is a serious challenge desiderated to be done. On the one hand, ionic liquids comprising of bulk organic cations and different anions attract significant interest as extremely promising cationic antibacterials. Long-chain ILs are efficient antibacterial agents against a wide range of clinical pathogenic microbial cultures that opens us to broader opportunities for their use as potential medical supplies [4,5,6]. On the other hand, the enzymes involved in bacterial fatty acid biosynthesis (FASII) are important targets for new antimicrobials [7]. This pathway is common for both gram-negative and gram-positive pathogens, e.g., *A. baumannii* and *S. aureus*, respectively. Enoyl-ACP reductase (FabI) (EC: 1.3.1.9) is one of the key enzymes in bacterial fatty acid biosynthesis (Figure 1) [8]. FabI catalyzes the reduction of the double bond in the enoyl moiety, which is connected to the acyl carrier protein (ACP). The enzyme also takes part in the fatty acids elongation cycle, which is important in lipid metabolism and biotin biosynthesis. In a number of studies, the mechanism of IL action is associated with AChE inhibition (EC_50_ values as low as 13 μM) [9]. The ILs inhibited AMP deaminase with IC_50_ ranged from 0.3 to 500 μM [10]. The inhibitory effect of IL on tyrosinase was also presented [11]. The interaction of lactic dehydrogenase with IL is known [12]. Imidazole and pyridine-containing compounds are one class of the known FabI inhibitors [13,14].

In this work, we analyzed a series of imidazolium and pyridinium based ionic liquids as potential FabI inhibitors with high antibacterial potential against MDR *A. baumannii* and *S. aureus* using QSAR modeling and biological tests.

## 2. Results

### 2.1. Regression Models (Dataset I)

The initial dataset of 1078 compounds with activity against *A. Baumannii* was split by chance into training (862) and test (216) sets. Since the activity data were collected from different sources, it contributed to their variation due to differences in the laboratory protocols used in different studies. The regression models built by the Trans-CNN [16], ASNN [17], and XGBOOST [18] methods (see Table 1) calculated the best performances. For this analysis E-state [19], ALOGPS [20], CDK2 [21], and Dragon descriptors [22] were included in the best models for all methods. The results are partially summarized in Table 1. Other statistical parameters and performances of individual models are shown in Figure S1 of the Supplementary Materials.

The q*^2^* values were 0.66–0.75 and 0.68–0.79 for training and test sets, respectively. A consensus model, which is an average of all four models, obtained the best performance. It was used to provide a quantitative evaluation of activity of compounds against *A. Baumannii* as described in Section 2.3. The variances of individual predictions of the consensus model were used to calibrate the prediction errors and estimate their applicability domain [23].

### 2.2. Regression Models (Dataset II)

We randomly divided the initial dataset of 212 compounds with activity against *S.* a*ureus* into a training set (164 compounds) and a test set (48 compounds). The models were developed using the same protocols as in Section 2.1. The QSAR models built by ASNN and RFR methods (see Table 2) calculated the highest performance. The final set of descriptors included E-state [19] and ALOGPS [20], CDK2 [21], and the type of anion, which systematically contributed to the top performing models for the investigated machine learning methods.

The selected models had similar performances in terms of R^2^, q^2^, and RMSE as summarized in Table 2 (see also Figure S2 of the Supplementary Materials). The calculated cross-validated coefficients were q^2^ = 0.70–0.77 for the training sets and q^2^ = 0.69–0.74 for the test sets. A consensus model, which was built by an average of these individual models, gave the best performance. It was used to provide a quantitative evaluation of potential anti-*S. Aureus* agents as described in the next section.

### 2.3. Evaluation of the Mode of Action (MoA) and Descriptor Importance

To investigate the influence of descriptor selection we developed ASNNs using E-state [19] and ALOGPS [20] descriptors, which contributed to the majority of models in Table 1 and Table 2. Usually in the OCHEM the ASNN models were developed with unsupervised selection of descriptors. To evaluate the importance of the descriptors, we used a sensitivity analysis method based on pruning algorithms [25,26] as implemented in the ASNN software. The pruning algorithms operate so-called “sensitivities” (S) [25,26] that are based on the evaluation of the importance of the weight matrix of the ANN. The pruning works similarly to a stepwise multiple regression analysis, whereby one input parameter considered to be non-significant is excluded at each step until performance of the model starts to decrease. Contrary to unsupervised descriptor selection, the pruning requires information about the target property as well as more computational resources.

Table S1 summarized the statistical parameters for models developed with merged set of descriptors selected for each property using unsupervised filtering and descriptor sets optimized using pruning. The pruning did not influence the performances of the respective models for both properties. Majority of descriptors, namely, 13 out of 15 descriptors for *S. aureus* were amid 27 descriptors selected for *A. Baumannii* (see Table S2). The number of descriptors selected for *A. Baumannii* was larger due to its higher diversity while the descriptor set for *S. Aureus* had SssssSi (atom-type E-state index for >Si< group), which was found for this set only. If we used descriptors selected for *A. Baumannii* and to develop model for *S. aureus,* we got model with very similar performance to that using descriptors selected specifically for *S. aureus*. This result can be used as indirect evidence that both properties share the same MoA. Contrary to that using 15 descriptors selected for *S. aureus* to develop model against *A. Baumannii* resulted in a model with much lower performance. Such lower performance could be due to insufficient diversity of the smaller *S. aureus* set.

### 2.4. Evaluation Activity of New Compounds

A virtual database of drug-like ILs was generated based on available synthetic blocks and reactions. It included 24 ILs with different substitution patterns (see Supplementary Materials, Table S3). These compounds were screened using the consensus model against *A. baumannii.* The 19 ILs predicted as most active within the applicability domain (i.e., compounds with Minimum Inhibitory Concentration MIC, <50 μM) were selected for further evaluation (see also Supplementary Materials, Table S4) and were screened using the consensus regression model against *S. aureus.* The 7 ILs with the highest predicted activity (MIC < 50μM) were retained for synthesis and testing (see Table 3 and Tables S3 and S4 in Supplementary Materials).

The next analysis was to examine the toxic effects of the studied ILs using the OCHEM published models against in vivo toxicity [27] and Ames test [28]. All seven compounds were predicted as inactive against the Ames test. The Table S5 demonstrates that drugs and tested ILs had similar toxicities associated with the species type, route of administration and toxicity types.

We have provided an analysis of the toxicity of ILs according to the OECD Guidance document on acute oral toxicity testing [29]. The Table S6 shows that the predicted oral LD_50_ values of compounds 3 and 4 are in the range of 188–310 mg/kg for all animal species, which corresponds to Category 3 of Globally Harmonized System of Classification and Labeling of Chemicals (GHS) while compounds 13, 16, 17, 20, and 22 with LD_50_ values ranging 317–1900 mg/kg belong to Category 4 of GHS severity.

The Table S7 shows that the predicted dermal LD_50_ values of compounds 3, 4, 20, and 22 ranging 410–917 mg/kg to rabbit belong to Category 3 of GHS according to the OECD Guidelines for the testing of chemicals [30]. However the predicted dermal toxicity (LD_50_) results of ILs 16 and 17 to rabbit and LD_50_ values of ILs 3 and 4 to rat ranging 1320–1560 mg/kg allow to classify them into Category 4. Compound 13 with predicted dermal LD_50_ value 2080 mg/kg to rabbit and compounds 13, 16, 17, 20, and 22 with predicted dermal LD_50_ values in the range of 2300–4990 mg/kg to rat belong to the Category 5 of severity.

Thereby, the comparative analysis of the predicted toxicity values of drugs and tested compounds allows classification of the ILs into the non-toxic Category 5 as well as to less toxic GST Categories 3 and 4, which indicates their promise as effective antimicrobials.

### 2.5. Biology

#### Antibacterial Activity

The biological study results of imidazolium and pyridinium based ionic liquids with predicted activity against MDR *A. baumannii* and *S. aureus* are shown in Table 3.

As shown in Table 3, compounds 3, 4, and 16 demonstrated the highest activity against antibiotic-resistant strains *A. baumannii* and *S. aureus* with inhibition zone diameters ranged from 16.3 to 20.3 mm and 19.7 to 25.3 mm, respectively. Their inhibitory activity was thus higher than that of known antibiotics Ceftriaxone, as well as Ampicillin and Oxacillin, which did not demonstrate any activity against these bacteria.

Considering that active compounds 3, 4, and 16 showed activity against both strains, we hypothesized that they shared a similar mechanism of action. Since imidazole and pyridine-containing compounds are known FabI inhibitors [13,14] and our active compounds contained these moieties, they could also inhibit this enzyme. We performed molecular docking and found a set of favorable binding poses and Gibbs energy estimations to support our idea (see Supporting Information for details). This study as well as descriptor sensitivities analysis both suggested that ILs may share the same MoA. However, the action mechanism could also be non-specific conditioned on the ability of long-chain moieties to incorporate into cell membranes [31]. Further experimental validations are required to answer this question.

## 3. Discussion

We used Online Chemical Modeling Environment to identify ionic liquids as antibacterials against MDR clinical isolate *A. baumannii* and *S. aureus* strains. The created QSAR models show high stability, robustness, and predictive power. The predictive accuracy of regression models had a coefficient of determination q^2^ = 0.66–0.79 with cross-validation and independent test sets. The sensitivity analysis identified common sets of descriptors, thus suggesting that the developed models predicted similar MoAs. The created models were successful to screen a virtual chemical library of ILs, which was designed with targeted activity against MDR *A. baumannii* and *S. aureus* strains.

Seven most promising ILs were identified, synthesized and tested against MDR *A. baumannii* and *S. aureus* strains. Three ILs, 1-dodecylpyridinium chloride (PyrC_12_-Cl), 1-tetradecylpyridinium bromide (PyrC_14_-Br) and 1-(2-hydroxyethyl)-3-dodecylimidazolium chloride (IMC_2_OHC_12_-Cl), demonstrated high activity against both these MDR clinical isolates.

Molecular docking of these ILs suggested that they can form a complex with FabI which is a key enzyme in the biosynthesis of bacterial fatty acids and could be a promising target for potential antibacterial drugs [32,33]. Our work also included the results of comparing the primary and secondary structures of the enzyme involved in the fatty acids metabolism of both bacterial pathogens, i.e., the significant structural enzyme similarity of AFabI and SFabI as shown in Figures S3 and S4 confirmed the qualitative visual similarity of the secondary structure of the studied enzymes.

It is important that among the wide range of known FabI inhibitors, there are also imidazole and pyridine derivatives, structurally similar to ILs, which were the object of our study [13]. Moreover, the success of using this enzyme as a target for antibacterials is due to the presence of certain structural features characteristic for bacterial biosynthesis of fatty acids (type II) in contrast with mammalian biosynthesis of fatty acids (type I).

However, considering that docking of ILs is a rather new application of the method, which has not been intensively validated with experimental studies, we cannot completely exclude other MOAs, such as ability of long-chain moieties to incorporate into cell membranes [31], could also explain the antibacterial activity of ILs. The future experimental studies using NMR and/or X-ray should provide a definite answer to this question.

The obtained results of biological testing confirmed the high antibacterial potential of the studied ILs (with high predicted activity) against both the gram-positive MDR clinical isolate *Staphylococcus aureus* and the gram-negative MDR clinical isolate *Acinetobacter baumannii* strains. The established fact is of special interest. Moreover, a number of authors [31,34] also presented experimental results the IL with longer alkyl chain had antibacterial activity against a number of gram-positive and gram-negative strains from the ATCC (American Type Culture Collection).

Our results not only allowed us to ascertain the high antibacterial potential of a number of long-chain ILs based on imidazolium and pyridinium against *Acinetobacter baumannii* (gram-negative bacteria) and *Staphylococcus aureus* (gram-positive bacteria) but also fulfill the promise of using studied ILs against their MDR clinical isolates.

## 4. Materials and Methods

### 4.1. Data

The data for our analysis were obtained from multiple publications. These data were uploaded into the On-line Chemical Database and Modeling Environment (OCHEM) [35] web-based platform designed for storing experimental properties and chemical activities with the primary goal of *in silico* modeling.

Two datasets were used to build the models against both analyzed strains. The first dataset (1078 compounds) consisted of diverse chemical series with minimum inhibitory concentration (MIC) values of the molecules ranging from 0.0906 to 9400 μM against *A. baumannii.* However, this set contained only few ILs. The second dataset included 212 ILs and their bioactivities against *S.* a*ureus*. The MIC values were ranging from 0.005 to 8600 μM. The log(1/MIC) values were used to develop regression models. By using models developed with both these sets we expected to design compounds active against both strains but also covering ILs.

### 4.2. On-Line Chemical Database and Modeling Environment

Interactive OCHEM web platform was used for creation public and freely accessible on-line models. The OCHEM calculates standard mean errors for each prediction and allows users to conclude whether the predictions are sufficiently accurate for their studies.

#### 4.2.1. Methods

Different machine-learning methods such as Associative Neural Networks (ASNNs) [17], Transformer Convolutional Neural Network (Trans-CNN) [16], XGBOOST [18], and Random Forest Regression (RFR) [24] were used to build QSAR models.

#### 4.2.2. Associative Neural Network (ASNN)

ASNN [17] represents a combination of an ensemble of the Feed-Forward Backpropagation Neural Networks and the k-Nearest Neighbors (kNN) method, which was inspired by thalamo-cortical organization of brain [36]. An ensemble of neural networks (n = 100) was used in this study while kNN provided its local correction to increase its accuracy.

#### 4.2.3. Extreme Gradient Boosting (XGBoost) 

XGBoost [18] is a machine learning algorithm based on a decision tree and using a gradient boosting framework. Gradient boosting is a learning technique for classification and regression problems that builds a prediction model in the form of an ensemble of weak predictive models, usually decision trees. The overfitting problem is solved by minimizing the norm of the learn weights. A greedy search is used to add new branches that most improve the objective function of the algorithm [18].

#### 4.2.4. Transformer Convolutional Neural Network (Trans-CNN)

The Trans-CNN method uses the internal representation of molecules based on their SMILES notation for extracting information-rich real-value embeddings during the encoding process and uses them for further QSAR-oriented blocks to model biological activity or physicochemical properties [16]. The Transformer-CNN architecture usually requires a few tens iterations to converge for new tasks. The method predicts the target value based on an average of an individual forecast for a batch of augmented SMILES belonging to the same molecule. The deviation within the batch can serve as a measure of a confidence interval of the prognosis, whereas the possibility to canonize SMILES can be used for deriving applicability domains of models.

#### 4.2.5. Random Forest Regression (RFR)

The random forest is a recursive partition ensemble method consisting of a number of decision trees. The decision tree is built using an initial copy of the training set and randomly selected subsets of descriptors. The final prediction is done by most of the votes of the individual trees [24].

#### 4.2.6. Descriptors

There are many software packages for the calculation of diverse types of molecular descriptors in the OCHEM. In this study, we used E-state indices [19], AlogPS [20], CDK2 [21], and Dragon descriptors [22] which were frequently top-performing descriptors according to our previous studies. Type of IL anion was provided as an additional descriptor using conditions of experiments feature of the OCHEM.

The electro-topological state indices define key structural features of a molecule and combine both electronic and topological attributes of the compounds [19].

AlogPS program calculates estimates lipophilicity (logP) and solubility in water (logS) of chemical compounds [20].

CDK calculates 256 molecular descriptors such as topological, geometrical, constitutional, electronic, and hybrid descriptors [21].

The Dragon 7 program calculates 5270 molecular descriptors. The user can calculate the simplest atom types, functional groups, fragment counts, several topological and geometrical descriptors, polar surface area, RDF, WHIM descriptors, atom-centered fragments, molecular properties, and many others [22]. The 3D structures of molecules were optimized using Corina [37].

#### 4.2.7. Descriptor Preprocessing

The unsupervised filtering of descriptors was used. Descriptors with fewer than two unique variables or with a coefficient of variance, less than 0.01 were omitted to avoid useless redundancy. Descriptors with values that tightly correlated with those of other descriptors (i.e., a pairwise non-parametric Pearson’s correlation coefficient R > 0.95) were grouped. Additionally, the Unsupervised Forward Selection (UFS) method [38] was applied to select a representative non-redundant set of descriptors. The selected descriptors were normalized to [−1, 1] interval for the ASNN but were used “as is” for RFR and XGBOOST methods, which are decision trees and do not require standardization of descriptors.

#### 4.2.8. Model Validation

Two validation protocols were used. First of all, the initial data were split by chance into training and test sets. For the training set five-fold cross-validation (CV) with variable selection in each step of the analysis was used to estimate accuracy of models for the training set [23]. Each data set of compounds was divided into 5 subsets of approximately equal size. Out of 5 subsets a single subset was retained for validation while the remaining data were used as training set. For each subdivision, OCHEM first selects descriptors using the respective training set, develops the model and then applies it to predict the excluded molecules from the respective validation set. Then the statistical coefficients for the full set combining all five test subsets were computed. The prediction performance of the final model was tested by using an external test set of compounds. The CV and results for prediction of the test set are reported.

Prediction accuracy estimation: The OCHEM incorporated a number of tools for estimation the applicability domain and the accuracy for each prediction [39]. The prediction accuracy is calibrated according to the performance of models during the CV analysis as described elsewhere [39].

We used two criteria to access the goodness of fitting: the squared correlation coefficient R^2^ and the coefficient of determination q^2^. In addition, we used root mean square error (RMSE) and the Mean Absolute Error (MAE) statistics to estimate the errors in predictions. A detailed description of used machine-learning methods, all selected descriptors, and validation procedures can be found in the Supplementary materials and in the OCHEM manual (http://docs.ochem.eu/display/MAN).

### 4.3. Synthesis

#### 4.3.1. General

Following chemicals were used for the synthesis of ionic liquids: pyridine (99%), 1-chlorododecane (97%), 1-bromotetradecane (97%), imidazole, 1-methylimidazole (for synthesis), chloroacetyl chloride (98%), 1-dodecanol (99%), 2-chloroethanol (99%), benzene, hexane, ethyl acetate (98%), methylene chloride (99%), thiourea (for synthesis), methanol (98%), hydrochloric acid (37%), isopropanol (98%), dodecylamine (for synthesis), (Sigma-Aldrich, Merck KGaA, St. Louis, MO, USA), 2-imidazolidinethione (98%) (Fluka, Thermo Fisher Scientific, Waltham, MA, USA). ^1^H NMR spectra were recorded in CDCl_3_ and DMSO-d_6_ on a 400 MHz Gemini-2000 (Varian, Inc., Palo Alto, CA, USA) spectrometer using TMS (tetramethyl silane) as internal standard. Melting points were determined on a Fisher–Johns (Thermo Fisher Scientific, Waltham, MA, USA) apparatus and are uncorrected.

#### 4.3.2. Synthesis of Ionic Liquids

**Scheme 1:**
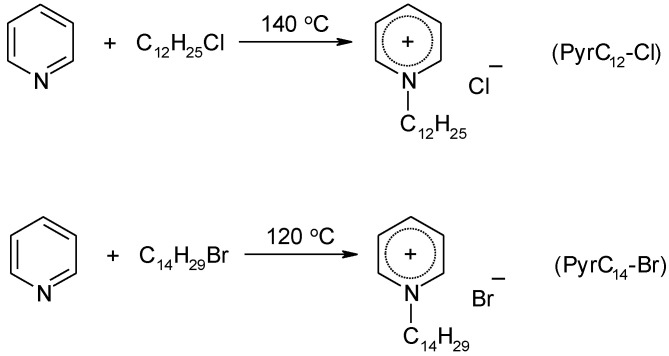
Synthesis of 1-alkylpyridinium ILs.

1-alkylpyridinium ionic liquids were synthesized according to Scheme 1.

1-dodecylpyridinium chloride (PyrC_12_-Cl) (**3**)

The mixture of pyridine (10 g, 0.12 mol) and 1-chlorododecane (31 g, 0.15 mol) was stirred at 140 °C for 20 h. The obtained solid product was purified by recrystallization from ethyl acetate-hexane mixture (1:3 v/v). Yield: 65% (22 g), white solid, mp 92–94 °C.

^1^H NMR (400 MHz, DMSO-D_6_): δ = 0.84 (t, 3H, CH_3_), 1.24 (m, 18H, CH_3_(CH_2_)_9_), 1.9 (m, 2H, NCH_2_CH_2_), 4.6 (t, 2H, NCH_2_), 8.2 (t, 2H, C_3_-H, C_5_-H), 8.6 (t, 1H, C_4_-H), 9.2 (d, 2H, C_2_-H, C_6_-H).

1-tetradecylpyridinium bromide (PyrC14-Br) (**4**)

The mixture of pyridine (10 g, 0.12 mol) and 1-bromotetradecane (36 g, 0.13 mol) was stirred at 120 °C for 2 h. The obtained solid product was purified by double recrystallization from ethyl acetate-hexane mixture (1:1 *v*/*v*). Yield: 76% (32 g), white solid, mp 64‒66 °C.

^1^H NMR (400 MHz, DMSO-D_6_): δ = 0.83 (t, 3H, CH_3_), 1.21 (m, 22H, CH_3_(CH_2_)_11_), 2.0 (m, 2H, NCH_2_CH_2_), 4.9 (t, 2H, NCH_2_), 8.14 (t, 2H, C_3_-H, C_5_-H), 8.51 (t, 1H, C_4_-H), 9.44 (d, 2H, C_2_-H, C_6_-H).

**Scheme 2:**
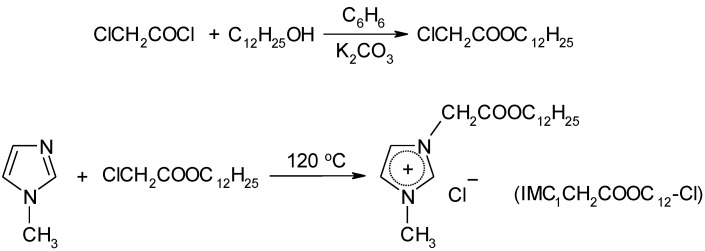
Synthesis of ester-functionalized imidazolium IL.

Long-chain imidazolium IL containing polar ester group in the alkyl radical was synthesized according to Scheme 2.

1-(dodecyloxycarbonylmethyl)-3-methylimidazolium chloride (IMC1CH2COOC12-Cl) (**13**)

Chloroacetyl chloride (13.5 g, 0.12 mol) was added dropwise to the stirred mixture of 1-dodecanol (20 g, 0.1 mol) and potassium carbonate (14 g, 0.1 mol) in dry benzene (200 mL). The reaction was carried out for 6 h at room temperature. After completion of the reaction, the reactionary mixture was washed with water until the pH became neutral. The organic layer was separated and dried overnight over calcium chloride. Benzene was distilled, residual solvent was removed in vacuum 10 mbar at 50 °C. The prepared crude dodecyl chloroacetate was further used for the synthesis of the IL.

The mixture of 1-methylimidazole (5 g, 0.06 mol) and dodecyl chloroacetate (18 g, 0.07 mol) was stirred at 120 °C for 2 h. After cooling, the solid product was purified by double recrystallization from ethyl acetate. Yield: 68% (14 g), white solid, mp 58–60 °C.

^1^H NMR (400 MHz, CDCl_3_): δ = 0.86 (t, 3H, CH_3_), 1.24 (m, 18H, (CH_2_)_9_), 1.63 (m, 2H, COOCH_2_CH_2_), 4.06 (s, 3H, NCH_3_), 4.16 (t, 2H, COOCH_2_), 5.45 (s, 2H, NCH_2_CO), 7.47 (d, 1H, C_4_-H), 7.54 (d, 1H, C_5_-H), 10.24 (s, 1H, C_2_-H).

**Scheme 3:**
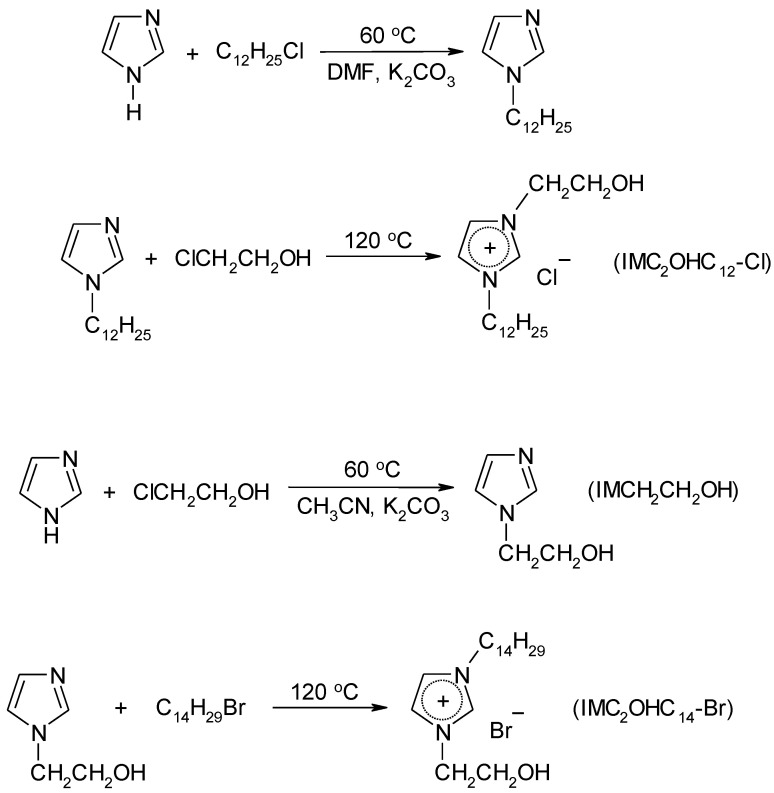
Synthesis of hydroxyl-functionalized imidazolium ILs.

Long-chain imidazolium ILs comprising polar 2-hydroxyethyl groups were synthesized according to Scheme 3.

1-(2-hydroxyethyl)-3-dodecylimidazolium chloride (IMC_2_OHC_12_-Cl) (**16**)

Potassium carbonate (38 g, 0.28 mol) was added to the solution of imidazole (12 g, 0.16 mol) and 1-chlorododecane (24 g, 0.12 mol) in 100 mL DMF. The mixture was stirred at 60 °C for 20 h after that it was poured into water (300 mL). The top layer of water immiscible oil product was separated, dissolved in methylene chloride (200 mL) and washed again with water (2 × 300 mL). The solution was dried over sodium sulfate. Methylene chloride was distilled and residual solvent was removed in vacuum 15 mbar at 60 °C. 1-dodecylimidazole was obtained as light yellow liquid.

^1^H NMR (400 MHz, CDCl_3_): δ = 0.87 (t, 3H, CH_3_), 1.24 (m, 18H, (CH_2_)_9_, 1.75 (m, 2H, NCH_2_CH_2_), 3.9 (t, 2H, NCH_2_), 6.89 (d, 1H, C_4_-H), 7.04 (d, 1H, C_5_-H), 7.44 (s, 1H, C_2_-H).

The mixture of 1-dodecylimidazole (15 g, 0.06 mol) and 2-chloroethanol (7.6 g, 0.095 mol) was stirred at 120–130 °C for 24 h. The residual reagent was removed in vacuum 5 mbar at 80 °C. The prepared semi-solid product of light brown color was purified by washing with hexane-ethyl acetate mixture (1:3 (*v*/*v*). Residual solvents were removed in vacuum 15 mbar at 60 °C. Yield: 45% (8.5 g), white semi-solid.

^1^H NMR (400 MHz, DMSO-D_6_): δ = 0.85 (t, 3H, CH_3_), 1.24 (m, 18H, CH_3_(CH_2_)_9_), 1.79 (m, 2H, NCH_2_CH_2_), 3.72 (t, 2H, NCH_2_CH_2_OH), 4.16 (t, 2H, NCH_2_), 4.22 (t, 2H, NCH_2_CH_2_OH), 5.35 (m, 1H, OH), 7.78 (br s, 1H, C_4_-H), 7.81 (br s, 1H, C_5_-H), 9.27 (s, 1H, C_2_-H).

1-(2-hydroxyethyl)-3-tetradecylimidazolium bromide (IMC_2_OHC_14_-Br) (**17**)

**Scheme 4:**
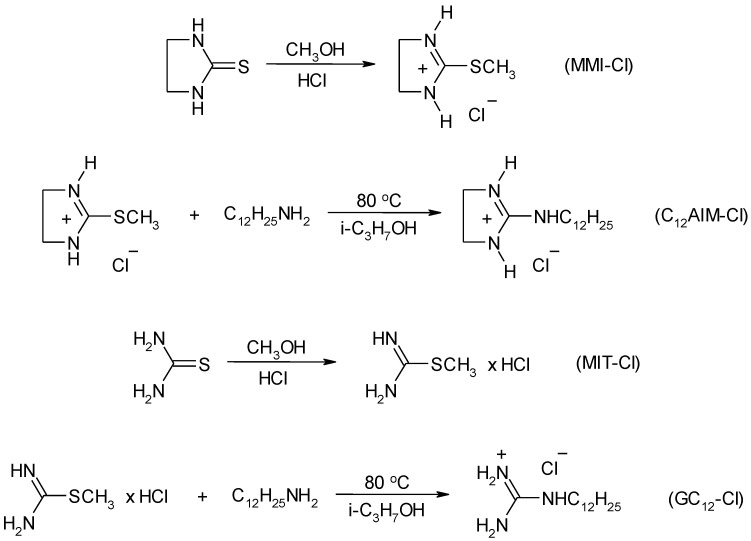
Synthesis of long-chain guanidinium based ionic liquids (ILs).

Potassium carbonate (38 g, 0.28 mol) was added to the solution of imidazole (10 g, 0.14 mol) and 2-chloroethanol (11 g, 0.14 mol) in 120 mL acetonitrile. The mixture was stirred at 60 °C for 20 h. The residue of inorganic salts was filtered off. Acetonitrile was distilled from reactionary mixture. Residual 2-chloroethanol was removed in vacuum 65 mbar at 80 °C. The crude product, 1-(2-hydroxyethyl)imidazole (Scheme 4) was prepared as viscous oil and used in the next stage without purification.

The mixture of crude 1-(2-hydroxyethyl)imidazole and 1-bromotetradecane (38 g, 0.14 mol) was stirred at 120 °C for 2 h. The obtained semi-solid product of light brown color was purified by washing with ethyl acetate (3 × 100 mL). Yield: 52% (28 g), white semi-solid.

^1^H NMR (400 MHz, DMSO-D_6_): δ = 0.85 (t, 3H, CH_3_), 1.23 (m, 22H, CH_3_(CH_2_)_11_), 1.78 (m, 2H, NCH_2_CH_2_), 3.73 (t, 2H, NCH_2_CH_2_OH), 4.18 (t, 2H, NCH_2_), 4.21 (t, 2H, NCH_2_CH_2_OH), 5.16 (m, 1H, OH), 7.77 (br s, 1H, C_4_-H), 7.81 (br s, 1H, C_5_-H), 9.2 (s, 1H, C_2_-H).

Guanidinium based long-chain ILs were synthesized according to Scheme 4.

2-dodecylaminoimidazoline-2 hydrochloride (C_12_AIM-Cl) (**20**)

2-methylmercaptoimidazoline-2 chlorohydrate was synthesized using the method described in [40]. 40 g (0.39 mol) of 2-imidazolidinethione were suspended in the mixture of methanol (50 mL) and concentrated hydrochloric acid (50 mL) with magnetic stirrer. The mixture was heated to reflux for 12 h. Residual methanol was distilled and water solution was evaporated. The solid residue was then purified by recrystallization from isopropanol. Yield: 70% (41.6 g), white solid, mp 160 °C.

^1^H NMR (300 MHz, DMSO-D_6_): δ = 2.71 (t, 3H, CH_3_), 3.84 (s, 4H, CH_2_), 10.64 (s, 2H, NH).

2-methylmercaptoimidazoline-2 chlorohydrate (15 g, 0.1 mol) was added to the stirred solution of dodecylamine (18.5 g, 0.1 mol) in 150 mL of isopropanol. The mixture was heated to boiling for 6 h. Methyl mercaptan released during the reaction was captured with 20% aqueous potassium hydroxide solution. Isopropanol was removed from the reactionary mixture at reduced pressure. The solid residue was purified by recrystallization from ethyl acetate-hexane (1:3 v/v) mixture. Yield: 74% (22 g), white solid, mp 54–56 °C.

^1^H NMR (300 MHz, DMSO-D_6_): δ = 0.85 (t, 3H, CH_3_), 1.25 (m, 18H, CH_3_(CH_2_)_9_), 1.46 (m, 2H, NCH_2_CH_2_), 3.18 (t, 2H, NCH_2_), 3.4 (s, 2H, 4-CH_2_), 3.56 (s, 2H, 5-CH_2_), 8.1–8.9 (br s, 3H, NH).

*N*-dodecylguanidine hydrochloride (C12G-Cl) (**22**)

S-methylisothiuronium chloride (MIT-Cl) was synthesized by the method similar to that described for the synthesis of 2-methylmercaptoimidazoline-2 chlorohydrate, using thiourea instead of ethylenethiourea. Yield: 65% (32 g), white solid, mp 116 °C. The ionic liquid C_12_G-Cl was prepared using the method similar to that described for the synthesis of C_12_AIM-Cl, using S-methylisothiuronium chloride instead of 2-methylmercaptoimidazoline-2 chlorohydrate (Scheme 4). After recrystallization from ethyl acetate-hexane (1:2 v/v) mixture, white solid residue was obtained. Yield: 65% (17 g), white solid, mp 116 °C.

^1^H NMR (400 MHz, DMSO-D_6_): δ = 0.86 (t, 3H, CH_3_), 1.25 (m, 18H, CH_3_(CH_2_)_9_), 1.46 (m, 2H, NCH_2_CH_2_), 3.11 (t, 2H, NCH_2_), 6.8–7.8 (br s, 4H, NH), 7.86 (s, 1H, NH).

### 4.4. Biology

The antibacterial activity of the studied ILs was estimated against MDR clinical isolates *A. baumannii* and *S. aureus.* The strains were provided from the Microbial Culture Collection Museum of the P.L. Shupyk National Medical Academy of Postgraduate Education.

Disc diffusion method in Mueller–Hinton agar was used to evaluate the antibacterial properties of studied compounds [41]. The inoculum was prepared at a final concentration of 1 × 10^5^ colony-forming unit (CFU) per mL using 0.5 McFarland standard as a reference to adjust the bacterial suspensions turbidity. Test compounds in an amount of 0.02 mL were applied to standard paper disks (6 mm), which were placed on each agar plate. All compounds were tested at identical concentrations and presented as content of compound on a disk that was 0.7 μM.

The known antibiotics Ampicillin, Oxacillin, and Ceftriaxone were used as positive controls.

The antibacterial activity of tested ILs was assessed by measuring zone diameter of the growth inhibition, which indicates the degree of susceptibility or resistance of *A. baumannii* and *S. aureus* isolate against the test compounds. The compounds, which formed zones > 15 mm of inhibition growth of microorganisms, were selected as active.

### 4.5. Molecular Docking

The docking was performed similarly to our earlier studies [42,43]. The crystal structures of enoyl-ACP reductase of *A. baumannii* (AFabI) and *S. aureus* (SFabI) were obtained from the RCSB Protein Data Bank (PDB ID: 6AH9, 3GR6) [44]. Subunits A of FabI *A. baumannii* and *S. aureus* were used for docking. The ligands and water molecules were deleted from the crystal structure using Accelrys DS 4.0 [45]. AutoDock Tools (ADT) 1.5.6 [46] was used to prepare the protein and ligands. All polar hydrogens were added to the protein molecules by ADT. The renumbering all atoms with included new hydrogen atoms were conducted by noBondOrder method. The Gasteiger method was applied for calculation and addition of partial charges. The prepared protein was saved in PDBQT format. The ChemAxon Marvin Sketch 5.3.735 program [47] was to create, optimize and save the ligand structures in Mol2 format. The ligands optimization and energy minimization were performed using MOPAC2016 [48] program. Partial charges and torsion angles of the ligands were altered with ADT and saved in PDBQT format. Docking was performed by AutoDock 4.2 [46] program. The Lamarckian genetic algorithm and rigid protein docking procedure were used for docking simulations. A grid and docking parameter files were generated by ADT tools. The ligand TCL (Triclosan) was set in the center of the box and the grid map (40 × 40 × 40 points) with grid spacing of 0.375Å. The other parameters were set as default. The analysis and visualization of protein-ligand interactions were performed by Accelrys DS 4.0 (Dassault Systemes, BIOVIA, Waltham, MA, USA).

## 5. Conclusions

A number of predictive regression models based on different machine learning methods were built using the OCHEM platform. The developed QSAR models demonstrated good stability, robustness, and predictive power. Seven ILs were synthesized and their activities against *A. baumannii* and *S. aureus* MDR isolates were evaluated. ILs **3**, **4**, and **16** with a long C12-C14 alkyl chain showed high in vitro activity against clinical isolates of both gram-positive *S. aureus* and gram-negative *A. baumannii* strains. These results are of certain interest due to the known data on ILs derived from N-cinnamyl imidazole with a similar alkyl chain length active only against gram-positive bacteria [31]. Molecular docking as well as a number of other indirect evidences demonstrated the ligand-FabI complexes formation could be the potential MoA, but this hypothesis need to be further validated experimentally.

## Figures and Tables

**Table 1:** Statistical coefficients calculated for QSAR models obtained for dataset I with activity against *A. baumannii*.

Method	Training Set ^a^			Test Set ^a^		
	R^2^	q^2^	RMSE ^c^	R^2^	q^2^	RMSE
ASNN ^b^	0.74 ± 0.04	0.73 ± 0.05	0.55 ± 0.04	0.74 ± 0.06	0.70 ± 0.08	0.64 ± 0.09
ASNN ^c^	0.70 ± 0.05	0.70 ± 0.05	0.59 ± 0.05	0.74 ± 0.06	0.69 ± 0.08	0.64 ± 0.09
RFR ^b^	0.73 ± 0.04	0.73 ± 0.04	0.55 ± 0.04	0.73 ± 0.09	0.72 ± 0.09	0.6 ± 0.1
Consensus ^d^	0.77 ± 0.04	0.77 ± 0.04	0.51 ± 0.04	0.77 ± 0.06	0.74 ± 0.07	0.6 ± 0.09

^a^ The training and test sets included 862 and 216 molecules, respectively. The cross-validation results are reported for the training set; ^b^ ASNN and XGBOOST models were developed by using E-state [19], ALOGPS [20] and CDK2 [21] descriptors; ^c^ ASNN model was created using Dragon [22] descriptors; ^d^ Consensus model was built by averaging outputs of all four models. R^2^—square of correlation coefficient; q^2^ coefficient of determination; RMSE—Root Mean Squared Error.

**Table 2:** Statistical coefficients calculated for QSAR models obtained for dataset II with activity against *S. aureus*.

Method	Training Set ^a^			Test Set ^a^		
	R^2^	q^2^	RMSE^c^	R^2^	q^2^	RMSE
ASNN ^b^	0.74 ± 0.04	0.73 ± 0.05	0.55 ± 0.04	0.74 ± 0.06	0.70± 0.08	0.64 ± 0.09
ASNN ^c^	0.70 ± 0.05	0.70 ± 0.05	0.59 ± 0.05	0.74 ± 0.06	0.69± 0.08	0.64 ± 0.09
RFR ^b^	0.73 ± 0.04	0.73 ± 0.04	0.55 ± 0.04	0.73 ± 0.09	0.72 ± 0.09	0.6 ± 0.1
Consensus ^d^	0.77 ± 0.04	0.77 ± 0.04	0.51 ± 0.04	0.77 ± 0.06	0.74 ± 0.07	0.6 ± 0.09

^a^ The training and test sets included 164 and 48 molecules, respectively. The cross-validation results are reported for the training set. ^b^ ASNN and RFR [24] models were developed by using E-state and ALOGPS descriptors; ^c^ ASNN model was created using CDK2 descriptors; ^d^ The consensus model was built by an average of the individual models.

**Table 3:** Antibacterial activities of studied ILs with predicted activity.

Compound	The Inhibition Zones Diameters (mm)	
	*A. baumannii*	*S. aureus*
3 *	20.3 ± 0.6	25.3 ± 0.6
4 *	17.0 ± 0.9	19.7 ± 0.3
13	15.3 ± 0.6	14.0 ± 0.6
16 *	16.3 ± 0.6	22.7 ± 0.9
17	17.0 ± 0.3	12.0 ± 0.3
20	15.7 ± 0.3	15.3 ± 0.3
22	11.0 ± 0.3	13.7 ± 0.6
Ampicillin	n/a	n/a
Oxacillin	n/a	n/a
Ceftriaxone	15.3 ± 0.3	15.7 ± 0.6

* Star indicates compounds with the highest activity. n/a—no activity. The standard mean errors were evaluated based on *n* = 3 experimental studies.

## Data Availability

All data and models are available at http://ochem.eu/article/125890.

## Supplementary Materials

Supplementary materials can be found at https://www.mdpi.com/1422-0067/22/2/563/s1.

## Abbreviations

QSAR	Quantitative Structure–Activity Relationship
OCHEM	Online Chemical Modeling Environment
ASNN	Associative Neural Network
XGBOOST	Extreme Gradient Boosting
Trans-CNN	Transformer Convolutional Neural Network
RFR	Random Forest Regression
kNN	k-Nearest Neighbors
RMSE	Root Mean Squared Error
R^2^	Square of correlation coefficient of determination
q^2^	Coefficient of determination
MDR	Multi-drug resistant
FASII	Bacterial fatty acid biosynthesis type II
ACP	Acyl carrier protein
AChE	Acetylcholinesterase
AMP	Adenosine monophosphate
NADP	Nicotinamide adenine dinucleotide phosphate
ADT	AutoDock Tools
TCL	Triclosan
FabI	Enoyl-ACP reductase
AFabI	Enoyl-ACP reductase A.baumannii
SFabI	Enoyl-ACP reductase S. aureus
ATCC	American Type Culture Collection

## References

[1] List of Bacteria for Which New Antibiotics Are Urgently Needed. https://www.who.int/news/item/27-02-2017-who-publishes-list-of-bacteria-for-which-new-antibiotics-are-urgently-needed.

[2] Coates R., Moran J., Horsburgh M.J. (2013). Staphylococci: Colonizers and pathogens of human skin. Future Microbiol..

[3] Martín-Aspas A., Guerrero-Sánchez F.M., García-Colchero F., Rodríguez-Roca S., Girón-González J.A. (2018). Differential characteristics of Acinetobacter baumannii colonization and infection: Risk factors, clinical picture, and mortality. Infect. Drug Resist..

[4] Trush M.M., Semenyuta I.V., Vdovenko S.I., Rogalsky S.P., Lobko E.O., Metelytsia L.O. (2017). Synthesis, spectroscopic and molecular docking studies of imidazolium and pyridinium based ionic liquids with HSA as potential antimicrobial agents. J. Mol. Struct..

[5] Miskiewicz A., Ceranowicz P., Szymczak M., Bartuś K., Kowalczyk P. (2018). The use of liquids ionic fluids as pharmaceutically active substances helpful in combating nosocomial infections induced by Klebsiella Pneumoniae new delhi strain, Acinetobacter Baumannii and Enterococcus species. Int. J. Mol. Sci..

[6] Ghanem O.B., Mutalib M., El-Harbawi M., Gonfa G., Kait C.F., Alitheen N.B.M., Lévêque J.M. (2015). Effect of imidazolium-based ionic liquids on bacterial growth inhibition investigated via experimental and QSAR modelling studies. J. Hazard. Mater..

[7] Wright H.T., Reynolds K.A. (2007). Antibacterial targets in fatty acid biosynthesis. Curr. Opin. Microbiol..

[8] Enoyl-[acyl-carrier-protein] Reductase (NADH). https://enzyme.expasy.org/EC/1.3.1.9.

[9] Stock F., Hoffmann J., Ranke J., Störmann R., Ondruschka B., Jastorff B. (2004). Effects of ionic liquids on the acetylcholinesterase—A structure–activity relationship consideration. Green Chem..

[10] Składanowski A.C., Stepnowski P., Kleszczyński K., Dmochowska B. (2005). AMP deaminase in vitro inhibition by xenobiotics: A potential molecular method for risk assessment of synthetic nitro- and polycyclic musks, imidazolium ionic liquids and N-glucopyranosyl ammonium salts. Environ. Toxicol. Pharmacol..

[11] Heitz M.P., Rupp J.W. (2018). Determining mushroom tyrosinase inhibition by imidazolium ionic liquids: A spectroscopic and molecular docking study. Int. J. Biol. Macromol..

[12] Dong X., Fan Y., Zhang H., Zhong Y., Yang Y., Miao J., Hua S. (2016). Inhibitory effects of ionic liquids on the lactic dehydrogenase activity. Int. J. Biol. Macromol..

[13] Heerding D.A., Chan G., DeWolf W.E., Fosberry A.P., Janson C.A., Jaworski D.D., McManus E., Miller W.H., Moore T.D., Payne D.J. (2001). 1,4-Disubstituted imidazoles are potential antibacterial agents functioning as inhibitors of enoyl acyl carrier protein reductase (FabI). Bioorg. Med. Chem. Lett..

[14] Desai N.C., Somani H., Trivedi A., Bhatt K., Nawale L., Khedkar V.M., Jha P.C., Sarkar D. (2016). Synthesis, biological evaluation and molecular docking study of some novel indole and pyridine based 1,3,4-oxadiazole derivatives as potential antitubercular agents. Bioorg. Med. Chem. Lett..

[15] Kanehisa M., Goto S. (2000). KEGG: Kyoto encyclopedia of genes and genomes. Nucleic Acids Res..

[16] Karpov P., Godin G., Tetko I.V. (2020). Transformer-CNN: Swiss knife for QSAR modeling and interpretation. J. Cheminform..

[17] Tetko I.V. (2008). Associative neural network. Methods Mol. Biol..

[18] Chen T., Guestrin C. (2016). XGBoost: A Scalable Tree Boosting System. arXiv.

[19] Hall L.H., Kier L.B. (1995). Electrotopological State Indexes for Atom Types—A Novel Combination of Electronic, Topological, and Valence State Information. J. Chem. Inf. Comput. Sci..

[20] Tetko I.V., Tanchuk V.Y. (2002). Application of associative neural networks for prediction of lipophilicity in ALOGPS 2.1 program. J. Chem. Inf. Comput. Sci..

[21] Willighagen E.L., Mayfield J.W., Alvarsson J., Berg A., Carlsson L., Jeliazkova N., Kuhn S., Pluskal T., Rojas-Cherto M., Spjuth O. (2017). The Chemistry Development Kit (CDK) v2.0: Atom typing, depiction, molecular formulas, and substructure searching. J. Cheminform..

[22] Todeschini R., Consonni V. (2000). Handbook of Molecular Descriptors.

[23] Tetko I.V., Sushko I., Pandey A.K., Zhu H., Tropsha A., Papa E., Oberg T., Todeschini R., Fourches D., Varnek A. (2008). Critical assessment of QSAR models of environmental toxicity against Tetrahymena pyriformis: Focusing on applicability domain and overfitting by variable selection. J. Chem. Inf. Model..

[24] Breiman L. (2001). Random forests. Mach. Learn..

[25] Tetko I.V., Villa A.E., Livingstone D.J. (1996). Neural network studies. 2. Variable selection. J. Chem. Inf. Comput. Sci..

[26] Kovalishyn V.V., Tetko I.V., Luik A.I., Kholodovych V.V., Villa A.E.P., Livingstone D.J. (1998). Neural network studies. 3. Variable selection in the cascade-correlation learning architecture. J. Chem. Inf. Comput. Sci..

[27] Sosnin S., Karlov D., Tetko I.V., Fedorov M.V. (2019). Comparative Study of Multitask Toxicity Modeling on a Broad Chemical Space. J. Chem. Inf. Model..

[28] Sushko I., Novotarskyi S., Körner R., Pandey A.K., Kovalishyn V.V., Prokopenko V.V., Tetko I.V. (2010). Applicability domain for *in silico* models to achieve accuracy of experimental measurements. J. Chemom..

[29] OECD (2001). OECD Guidance Document on Acute Oral Toxicity Testing. OECD Series on Testing and Assessment.

[30] OECD Guidelines for the Testing of Chemicals. https://www.oecd-ilibrary.org/environment/oecd-guidelines-for-the-testing-of-chemicals-section-4-health-effects_20745788.

[31] Doria O.F., Castro R., Gutierrez M., Valenzuela D.G., Santos L., Ramirez D., Guzman L. (2018). Novel alkylimidazolium ionic liquids as an antibacterial alternative to pathogens of the skin and soft tissue infections. Molecules.

[32] Yao J., Rock C.O. (2017). Bacterial fatty acid metabolism in modern antibiotic discovery. Biochim. Biophys. Acta.

[33] Massengo-Tiassé R.P., Cronan J.E. (2009). Diversity in enoyl-acyl carrier protein reductases. Cell. Mol. Life Sci..

[34] Neves Y.F., Eloi A.C.L., de Freitas H.M.M., Soares E.G.O., Rivillo D., Demétrio da Silva V., Schrekker H.S., Badel J.L. (2020). Imidazolium salts as alternative compounds to control diseases caused by plant pathogenic bacteria. J. Appl. Microbiol..

[35] Sushko I., Novotarskyi S., Korner R., Pandey A.K., Rupp M., Teetz W., Brandmaier S., Abdelaziz A., Prokopenko V.V., Tanchuk V.Y. (2011). Online chemical modeling environment (OCHEM): Web platform for data storage, model development and publishing of chemical information. J. Comput. Aided. Mol. Des..

[36] Villa A.E., Tetko I.V., Dutoit P., De Ribaupierre Y., De Ribaupierre F. (1999). Corticofugal modulation of functional connectivity within the auditory thalamus of rat, guinea pig and cat revealed by cooling deactivation. J. Neurosci. Methods.

[37] Sadowski J., Gasteiger J., Klebe G. (1994). Comparison of Automatic Three-Dimensional Model Builders Using 639 X-ray Structures. J. Chem. Inf. Comput. Sci..

[38] Whitley D.C., Ford M.G., Livingstone D.J. (2000). Unsupervised forward selection: A method for eliminating redundant variables. J. Chem. Inf. Comput. Sci..

[39] Sushko I., Novotarskyi S., Korner R., Pandey A.K., Cherkasov A., Li J., Gramatica P., Hansen K., Schroeter T., Muller K.R. (2010). Applicability domains for classification problems: Benchmarking of distance to models for Ames mutagenicity set. J. Chem. Inf. Model..

[40] Denk M.K., Ye X. (2005). Alkylation of ethylenethiourea with alcohols: A convenient synthesis of S-alkyl-isothioureas without toxic alkylating agents. Tetrahedron Lett..

[41] Bauer A.W., Kirby W.M., Sherris J.C., Turck M. (1966). Antibiotic susceptibility testing by a standardized single disk method. Am. J. Clin. Pathol..

[42] Metelytsia L., Hodyna D., Dobrodub I., Semenyuta I., Zavhorodnii M., Blagodatny V., Kovalishyn V., Brazhko O. (2020). Design of (quinolin-4-ylthio)carboxylic acids as new Escherichia coli DNA gyrase B inhibitors: Machine learning studies, molecular docking, synthesis and biological testing. Comput. Biol. Chem..

[43] Semenyuta I.V., Kobzar O.L., Hodyna D.M., Brovarets V.S., Metelytsia L.O. (2019). *In silico* study of 4-phosphorylated derivatives of 1,3-oxazole as inhibitors of Candida albicans fructose-1,6-bisphosphate aldolase II. Heliyon.

[44] Rose P.W., Bi C., Bluhm W.F., Christie C.H., Dimitropoulos D., Dutta S., Green R.K., Goodsell D.S., Prlić A., Quesada M. (2013). The RCSB Protein Data Bank: New resources for research and education. Nucleic Acids Res..

[45] Discovery Studio Visualizer Software, Version 4.0. https://www.3ds.com/biovia/.

[46] Trott O., Olson A.J. (2010). AutoDock Vina: Improving the speed and accuracy of docking with a new scoring function, efficient optimization, and multithreading. J. Comp. Chem..

[47] ChemAxon Marvin Sketch. http://www.chemaxon.com.

[48] Stewart J.J. MOPAC2016.

