# Supplementary material for: Structure-Activity Relationship Modeling and Experimental Validation of the Imidazolium and Pyridinium Based Ionic Liquids as Potential Antibacterials of MDR Acinetobacter baumannii and Staphylococcus aureus
Source: Int J Mol Sci. 2021 Jan 8;22(2):563. doi: 10.3390/ijms22020563 (PMC7827895; doi:10.3390/ijms22020563)
Supplement: Supplementary file 1 [file ijms-22-00563-s001.pdf]

Appendix

Structure-Activity relationship modeling and experimental validation of the imidazolium and pyridinium based ionic liquids as potential antibacterials of MDR *Acinetobacter baumannii* and *Staphylococcus aureus*

Ivan V. Semenyuta [1], Maria M. Trush [1], Vasyl V. Kovalishyn [1], Sergiy P. Rogalsky [1], Diana M. Hodyna [1], Pavel Karpov [2], Zhonghua Xia [2], Igor V. Tetko[2,3,*], Larisa O. Metelytsia[1]

[1] V.P. Kukhar Institute of Bioorganic Chemistry and Petrochemistry, National Academy of Science of Ukraine, 1 Murmanska Street, 02660, Kyiv, Ukraine; ivan@bpci.kiev.ua (I.V.S.); maria@bpci.kiev.ua (M.M.T); vkovalishyn@bpci.kiev.ua (V.V.K.); sergey.rogalsky@gmail.com (S.P.R.); hodyna@bpci.kiev.ua (D.M.H); metelitsa@bpci.kiev.ua (L.O.M.)

[2] Institute of Structural Biology, Helmholtz Zentrum München - German Research Center for Environmental Health (GmbH), Ingolstädter Landstraße 1, D-85764 Neuherberg, Germany; i.tetko@helmholtz-muenchen.de; (I.V.T.); pavel.karpov@helmholtz-muenchen.de (P.K.); zhonghua.xia@helmholtz-muenchen.de (Z.X.)

[3] BIGCHEM GmbH, Unterschleißheim, Valerystr. 49, D-85716, Germany; (I.V.T.)

* Correspondence: i.tetko@helmholtz-muenchen.de; Tel.: + 49-89-31873575

1.1 Computational Machine Learning results

Regression models from Table 1

Model name: M1_AcinBaum_MIC_TRANSNN_10/10 - 351229 [rename] , published in Structure-Activity relationship modeling and experimental validation of the imidazolium and pyridinium based ionic liquids as potential antibacterials of MDR Acinetobacter baumannii and Staphylococcus aureus
Public ID is 824

Predicted property: **AcinBaum_MIC** modeled in -log(M)
Training method: TRANSNN

Data Set	#	R2	q2	RMSE	MAE
o Training set: A_Baumanii_Set_II (training)	862 records	0.73 ± 0.02	0.73 ± 0.02	0.46 ± 0.02	0.32 ± 0.01
o Test set: A_Baumanii_Set_II (test) [x]	216 records	0.78 ± 0.03	0.78 ± 0.02	0.41 ± 0.02	0.29 ± 0.02

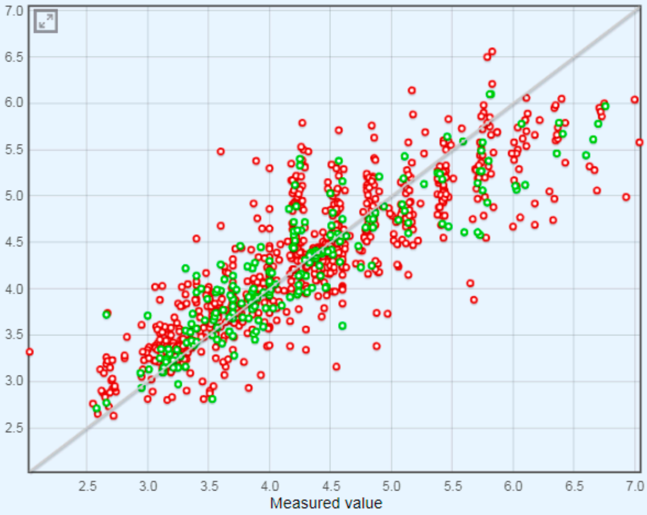

a)

Model name: M2_AcinBaum_MIC_ASNN_[ALogPS, CDK2 (constitutional, topological... - 364680 [rename] , published in Structure-Activity relationship modeling and experimental validation of the imidazolium and pyridinium based ionic liquids as potential antibacterials of MDR Acinetobacter baumannii and Staphylococcus aureus
Public ID is 825

Predicted property: **AcinBaum_MIC** modeled in -log(M)
Training method: ASNN

Data Set	#	R2	q2	RMSE	MAE
o Training set: A_Baumanii_Set_II (training)	820 records	0.7 ± 0.02	0.69 ± 0.03	0.48 ± 0.02	0.32 ± 0.01
o Test set: A_Baumanii_Set_II (test) [x]	207 records	0.69 ± 0.04	0.68 ± 0.05	0.49 ± 0.04	0.33 ± 0.02

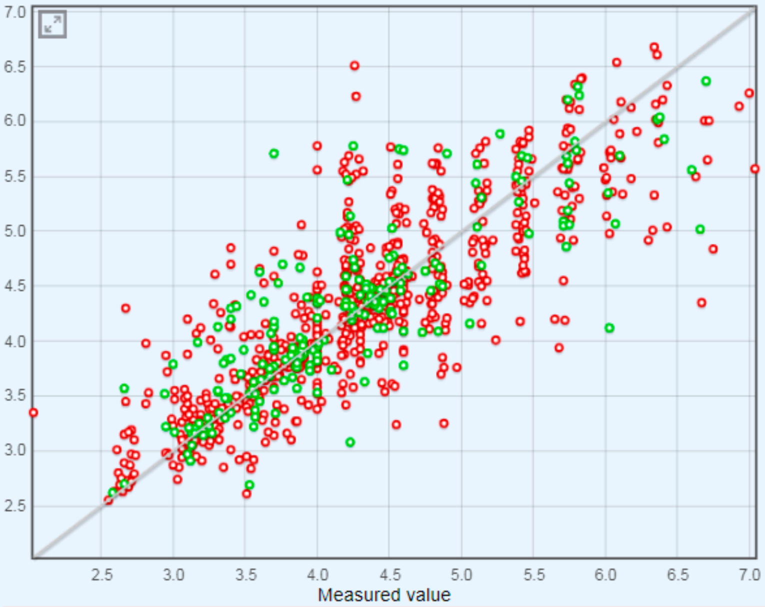

b)

Model name: M3_AcinBaum_MIC_ASNN_[Dragon7 (3D blocks: 1-30)] - 364719 [rename] , published in Structure-Activity relationship modeling and experimental validation of the imidazolium and pyridinium based ionic liquids as potential antibacterials of MDR Acinetobacter baumannii and Staphylococcus aureus
Public ID is 826

Predicted property: **AcinBaum_MIC** modeled in -log(M)
Training method: ASNN

Data Set	#	R2	q2	RMSE	MAE
o Training set: A_Baumanii_Set_II (training)	862 records	0.67 ± 0.02	0.66 ± 0.02	0.51 ± 0.02	0.37 ± 0.01
o Test set: A_Baumanii_Set_II (test) [x]	216 records	0.7 ± 0.04	0.7 ± 0.04	0.47 ± 0.03	0.35 ± 0.02

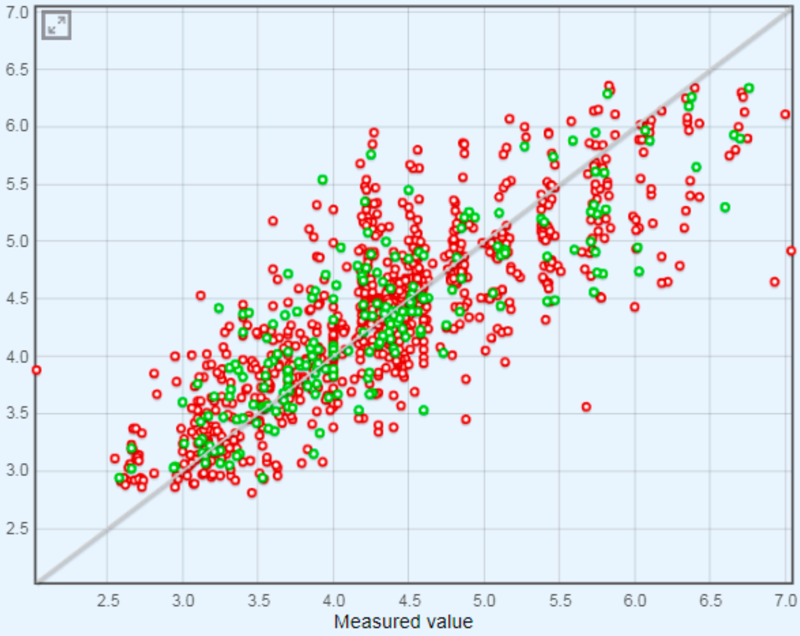

c)

Model name: M4_AcinBaum_MIC_XGBOOST_[ALogPS, CDK2 (constitutional, topologi... - 364720 [rename] , published in Structure-Activity relationship modeling and experimental validation of the imidazolium and pyridinium based ionic liquids as potential antibacterials of MDR Acinetobacter baumannii and Staphylococcus aureus
Public ID is 827

Predicted property: **AcinBaum_MIC** modeled in -log(M)
Training method: XGBOOST

Data Set	#	R2	q2	RMSE	MAE
o Training set: A_Baumanii_Set_II (training)	862 records	0.67 ± 0.02	0.67 ± 0.02	0.5 ± 0.02	0.35 ± 0.01
o Test set: A_Baumanii_Set_II (test) [x]	216 records	0.73 ± 0.03	0.73 ± 0.03	0.45 ± 0.03	0.31 ± 0.02

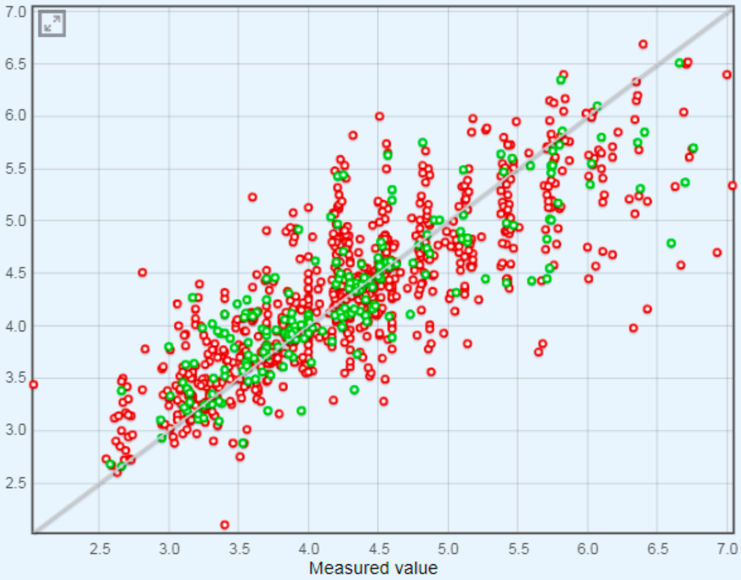

d)

Model name: M5_Consensus AcinBaum_MIC - 374840 [rename] , published in Structure-Activity relationship modeling and experimental validation of the imidazolium and pyridinium based ionic liquids as potential antibacterials of MDR Acinetobacter baumannii and Staphylococcus aureus
Public ID is 828

Predicted property: **AcinBaum_MIC** modeled in -log(M)
Training method: Consensus

Data Set	#	R^2	q2	RMSE	MAE
Training set: A_Baumanii_Set_II (training)	862 records	0.75 ± 0.02	0.75 ± 0.02	0.44 ± 0.02	0.31 ± 0.01
Test set: A_Baumanii_Set_II (test) [x]	216 records	0.8 ± 0.03	0.79 ± 0.03	0.4 ± 0.02	0.28 ± 0.02

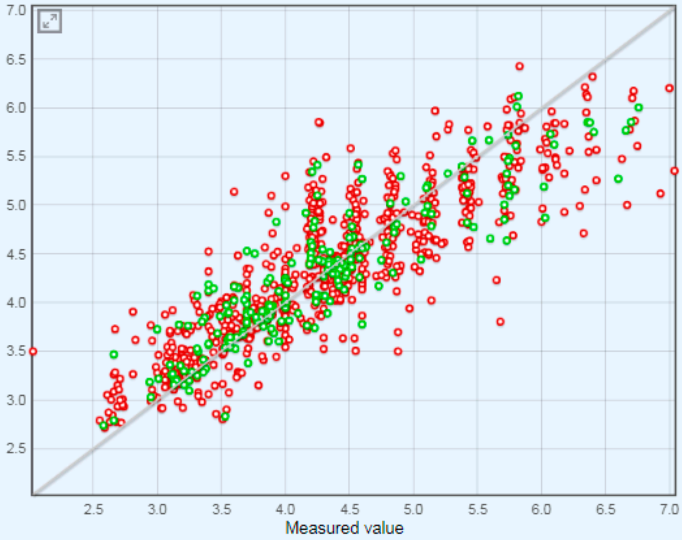

e)

Figure S1. QSAR models developed using the OCHEM [1] (http://ochem.eu). a-d) Statistical coefficients calculated for analyzed machine learning models; e) Consensus model calculated on the basis of four models.

Regression models from Table 2

Model name: M1_ASNN_[ALogPS, EState,Type of anion] - 364549 [rename] , published in Structure-Activity relationship modeling and experimental validation of the imidazolium and pyridinium based ionic liquids as potential antibacterials of MDR Acinetobacter baumannii and Staphylococcus aureus
Public ID is 829

Predicted property: **MIC** modeled in -log(M)
Training method: ASNN

Data Set	#	R2	q2	RMSE	MAE
Training set: Data_Set_S_Aureus (training)	164 records	0.74 ± 0.04	0.73 ± 0.05	0.55 ± 0.04	0.41 ± 0.03
Test set: Data_Set_S_Aureus (test) [x]	48 records	0.74 ± 0.06	0.7 ± 0.08	0.6 ± 0.1	0.45 ± 0.07

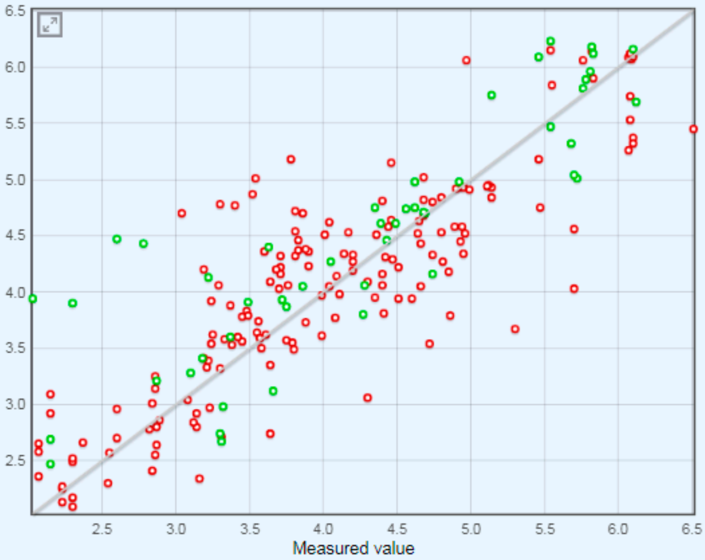

a)

Model name: M2_ASNN_[CDK2 (constitutional, topological, geometrical, electronic, hybrid)] - 365851 [rename] , published in Structure-Activity relationship modeling and experimental validation of the imidazolium and pyridinium based ionic liquids as potential antibacterials of MDR Acinetobacter baumannii and Staphylococcus aureus
Public ID is 830

Predicted property: **MIC** modeled in -log(M)
Training method: ASNN

Data Set	#	R2	q2	RMSE	MAE
Training set: Data_Set_S_Aureus (training)	142 records	0.7 ± 0.05	0.7 ± 0.05	0.59 ± 0.05	0.43 ± 0.03
Test set: Data_Set_S_Aureus (test) [x]	40 records	0.74 ± 0.06	0.69 ± 0.08	0.64 ± 0.09	0.47 ± 0.07

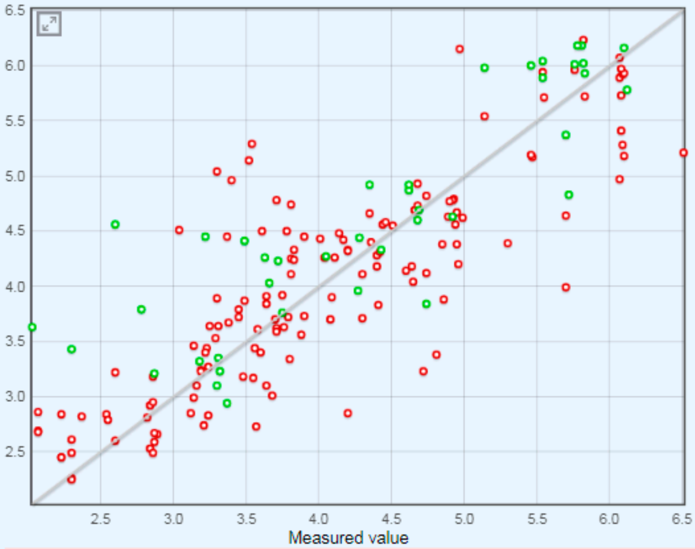

b)

Model name: M3_RFR_[ALogPS, EState,Type of Anion] - 364558 [rename] , published in Structure-Activity relationship modeling and experimental validation of the imidazolium and pyridinium based ionic liquids as potential antibacterials of MDR Acinetobacter baumannii and Staphylococcus aureus
Public ID is 831

Predicted property: **MIC** modeled in -log(M)
Training method: RFR

Data Set	#	R2	q2	RMSE	MAE
o Training set: Data_Set_S_Aureus (training)	162 records	0.73 ± 0.04	0.73 ± 0.04	0.55 ± 0.04	0.39 ± 0.03
o Test set: Data_Set_S_Aureus (test) [x]	45 records	0.73 ± 0.09	0.72 ± 0.09	0.6 ± 0.1	0.39 ± 0.07

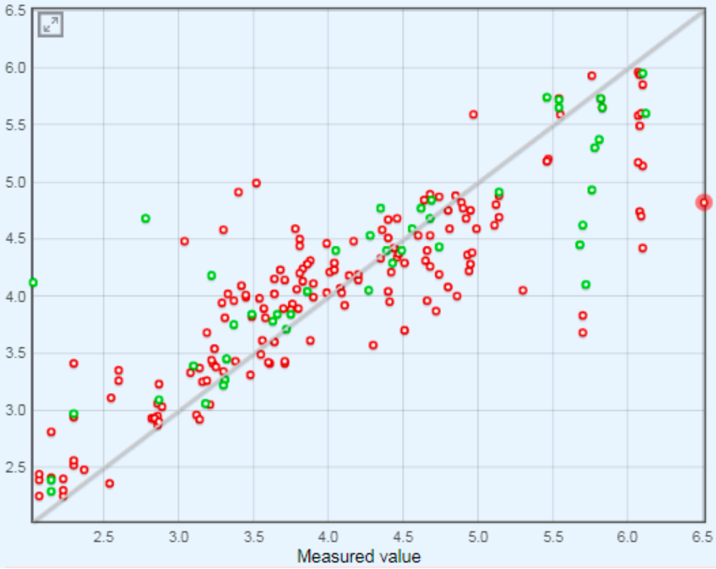

c)

Model name: M4_Consensus MIC - 374841 [rename] , published in Structure-Activity relationship modeling and experimental validation of the imidazolium and pyridinium based ionic liquids as potential antibacterials of MDR Acinetobacter baumannii and Staphylococcus aureus
Public ID is 832

Predicted property: **MIC** modeled in -log(M)
Training method: Consensus

Data Set	#	R2	q2	RMSE	MAE
o Training set: Data_Set_S_Aureus (training)	164 records	0.77 ± 0.04	0.77 ± 0.04	0.51 ± 0.04	0.37 ± 0.03
o Test set: Data_Set_S_Aureus (test) [x]	48 records	0.77 ± 0.06	0.74 ± 0.07	0.6 ± 0.1	0.39 ± 0.07

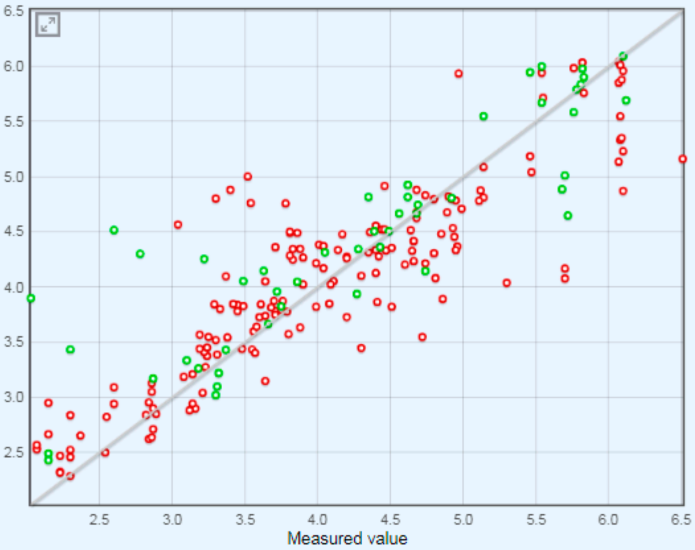

d)

Figure S2. QSAR models developed using the OCHEM [1] (http://ochem.eu). a-d) Statistical coefficients calculated for analyzed machine learning models; e) Consensus model calculated on the basis of three models.

1.2. Evaluation of the Mode of Action (MoA) and Descriptor Importance

Table S1. Statistical coefficients calculated for *A. Baumannii* and *S. Aureus* following descriptor selection with ASNN model

Target	Study	Number of descriptors	Training set			Test set		
			Q^2	RMSE	MAE	Q^2	RMSE	MAE
A. Baum.	Without pruning	38[a]	0.72 ± 0.01	0.47± 0.01	0.33	0.72± 0.01	0.46± 0.01	0.34
	Pruning of descriptors	27[b]	0.71± 0.01	0.47± 0.01	0.33	0.73± 0.01	0.45± 0.01	0.32
		15[c]	0.66± 0.01	0.51± 0.01	0.37	0.64± 0.01	0.52± 0.01	0.39
S. Aureus	Without pruning	38[a]	0.74 ± 0.01	0.54± 0.01	0.39	0.66± 0.01	0.69 ± 0.01	0.43
	Pruning of descriptors	15[c]	0.74± 0.01	0.53± 0.01	0.38	0.67± 0.01	0.68± 0.01	0.44
		27[b]	0.74± 0.01	0.55± 0.01	0.39	0.66± 0.01	0.69± 0.01	0.43

[a]Models based on the initial set of descriptors. [b]Models based on 27 descriptors selected using ***A. Baumannii***. [c]Models based on 15 descriptors selected using ***S. Aureus***. RMSE – Root Mean Squared Error; MAE - Mean Absolute Error; Q^2 – coefficient of determination.

Table S2. Molecular descriptors selected by pruning methods

No	*Acinetobacter Baumannii*	*Staphylococcus aureus*	Description
**1**		ALogPS_logP	octanol/water partition coefficient
**2**	ALogPS_logS	ALogPS_logS	solubility in water
**3**	SssCH2	SssCH2	Atom-type E-state index for -CH2- groups
**4**	SddssS	SddssS	Atom-type E-state index for >S== groups (sulfate)
**5**	SsssN	SsssN	Atom-type E-state index for >N- groups
**6**	SsH	SsH	Atom-type E-state index for -H groups
**7**	SaasN	SaasN	Atom-type E-state index for aaN- groups (e.g., substituted imidazole)
**8**	SaasC	SaasC	Atom-type E-state index for -Caa groups
**9**	SsOH	SsOH	Atom-type E-state index for -OH groups
**10**	SaaaC	SaaaC	Atom-type E-state index for aCaa groups
**11**	SssO	SssO	Atom-type E-state index for -O- groups
**12**	SsssCH	SsssCH	Atom-type E-state index for >CH- groups
**13**	SstC	SstC	Atom-type E-state index for #C- groups
**14**		SdO	Atom-type E-state index for =O- groups
**15**		SssssSi	Atom-type E-state index for >Si< groups
**16**	SssNH		Atom-type E-state index for -NH- groups
**17**	SddsN		Atom-type E-state index for -N== groups (nitro)
**18**	SaaO		Atom-type E-state index for aOa groups
**19**	SaadC		Atom-type E-state index for =Caa groups (e.g., C=O in theophylline)
**20**	SaaCH		Atom-type E-state index for aCHa groups
**21**	SdssC		Atom-type E-state index for =C= groups
**22**	SdS		Atom-type E-state index for =S- groups
**23**	SsF		Atom-type E-state index for -F groups
**24**	SssssC		Atom-type E-state index for >C< groups
**25**	SdsN		Atom-type E-state index for =N- groups
**26**	SaaNH		Atom-type E-state index for aNHa groups
**27**	SsNH2		Atom-type E-state index for -NH2 groups
**28**	SssS		Atom-type E-state index for -S- groups
**29**	SdNHC		Atom-type E-state index for =NHC groups
**30**	SdsCH		Atom-type E-state index for =CH- groups

[a]Descriptors selected for both bacteria using pruning methods from the common set of 38 descriptors.

1.3 *Predicted activity of new compounds*

Table S3. Structures of the 24 ILs analyzed in this work.

Compound No	Chemical Structure	Weight	Chemical Name
1	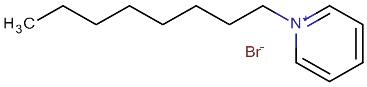	272.22	1-octylpyridinium bromide
2	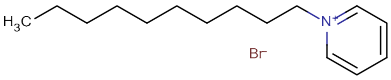	300.28	1-decylpyridinium bromide
3[a]	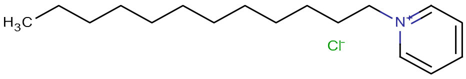	**283.88**	**1-dodecylpyridinium chloride**
4	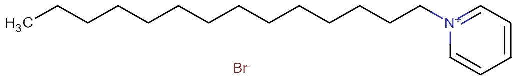	**356.38**	**1-tetradecylpyridinium bromide**
5	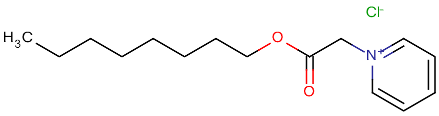	285.81	1-octyloxycarbonylmethylpyridinium chloride
6	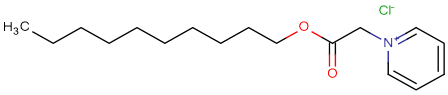	313.86	1-decyloxycarbonylmethylpyridinium chloride
7	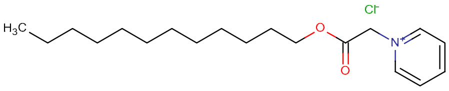	341.92	1-dodecyloxycarbonylmethylpyridinium chloride
8	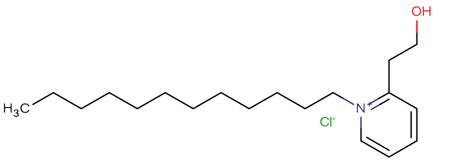	327.93	2-(2-hydroxyethyl)-1-dodecylpyridinium chloride
9	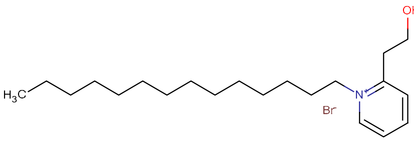	400.44	2-(2-hydroxyethyl)-1-tetradecylpyridinium bromide
10	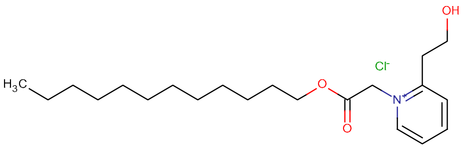	385.97	2-hydroxyethyl-1- dodecyloxy carbonylmethylpyridinium chloride
11	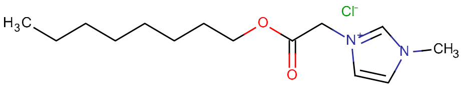	288.81	1-(octyloxycarbonylmethyl)-3-methylimidazolium chloride
12	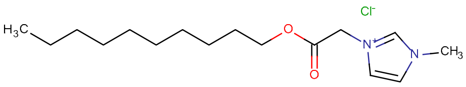	316.87	1-(decyloxycarbonylmethyl)-3-methylimidazolium chloride

**13**	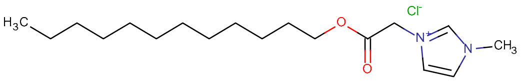	**344.92**	**1-(dodecyloxycarbonylmethyl)-3-methylimidazolium chloride**
14	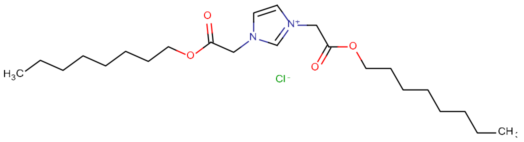	445.04	1,3-bis(octyloxycarbonylmethyl)imidazolium chloride
15	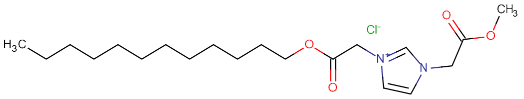	402.96	1-dodecyloxycarbonylmethyl-3-methyloxycarbonylmethylimidazolium chloride
**16**	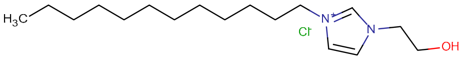	**316.91**	**1-(2-hydroxyethyl)-3-dodecylimidazolium chloride**
**17**	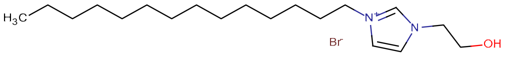	**389.41**	**1-(2-hydroxyethyl)-3-tetradecylimidazolium bromide**
18	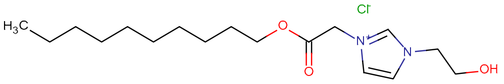	346.89	1-(2-hydroxyethyl)-3-decyloxycarbonylmethylimidazolium chloride
19	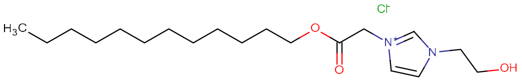	374.95	1-(2-hydroxyethyl)-3-dodecyloxycarbonylmethylimidazolium chloride
**20**	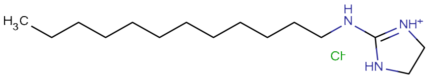	**289.89**	**2-dodecylaminoimidazoline hydrochloride**
21	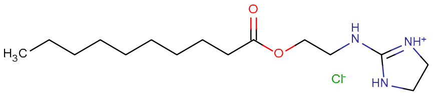	319.87	2-nonylcarbonyloxyethylamino imidazoline hydrochloride
**22**	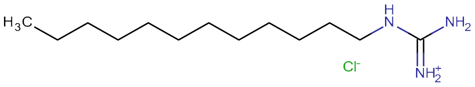	**263.85**	**N-dodecylguanidine hydrochloride**
23	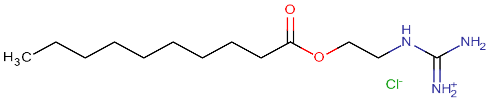	293.83	N-nonylcarbonyloxyethyl guanidine hydrochloride
24	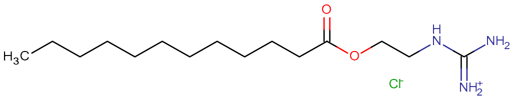	321.87	N-undecylcarbonyloxyethyl guanidine hydrochloride

[a]Final set compounds are represented in bold.

Table S4. Prediction of 24 ILs using consensus model developed against both bacteria

Comp. No.	*Acinetobacter Baumannii*			*Staphylococcus aureus*		
	log(1/MIC), M	RMSE	AD	log(1/MIC), M	RMSE	AD
1	3.9	0.5	TRUE			
2	4.4	0.5	TRUE	3.8	0.6	TRUE
**3[a]**	**4.4**	**0.5**	**TRUE**	**4.3**	**0.6**	**TRUE**
**4[a]**	**4.4**	**0.5**	**TRUE**	**4.3**	**0.6**	**TRUE**
5[b]	*3.9*	*0.4*	*TRUE*			
6[b]	*4*	*0.4*	*TRUE*			
7[b]	*4*	*0.5*	*TRUE*			
8	4.4	0.5	TRUE	4.4	0.6	FALSE
9[b]	*4.4*	*0.5*	*FALSE*			
10	4.1	0.5	TRUE	3.9	0.6	TRUE
11[b]	3.9	0.5	TRUE			
12	4.2	0.4	TRUE	3.9	0.6	TRUE
**13[a]**	**4.2**	**0.5**	**TRUE**	**4.1**	**0.6**	**TRUE**
14	4	0.5	TRUE	3.8	0.6	TRUE
15	4.2	0.5	TRUE	3.8	0.6	TRUE
**16[a]**	**4.2**	**0.5**	**TRUE**	**4.4**	**0.6**	**TRUE**
**17[a]**	**4.2**	**0.5**	**TRUE**	**4.4**	**0.6**	**TRUE**
18	4.2	0.5	TRUE	3.7	0.6	TRUE
19	4.2	0.5	TRUE	3.8	0.6	TRUE
**20[a]**	**4.6**	**0.5**	**TRUE**	**4.3**	**0.6**	**TRUE**
21	4.4	0.5	TRUE	3.6	0.6	FALSE
**22[a]**	**4.5**	**0.5**	**TRUE**	**4**	**0.6**	**TRUE**
23	4.3	0.4	TRUE	3.5	0.6	FALSE
24	4.5	0.5	TRUE	3.6	0.6	FALSE

[a]Final set compounds are represented in bold. [b]Compounds with the low activity or outside of the applicability domain that were filtered out following the application of ***Acinetobacter Baumannii*** model and thus were not considered for the ***Staphylococcus aureus*** model. MIC - minimum inhibitory concentration, MIC values are in mol/L; RMSE – predicted root mean square error; AD –applicability domain.

Table S5. Comparative analysis of toxicity predictions for drugs (DrugBank) and tested ILs using http://ochem.eu/multitox model [2].

No	Species	Administration route	Toxi-city endpoint	Average toxicity drugs	Tested ILs,						
					3	4	13	16	17	20	22
**1**	guinea pig	oral	LD_{50}	2.57	3.2	3.2	2.6	2.7	2.7	2.9	2.9
**2**	mammal, species	unreported	LD_{50}	2.64	3.6	3.6	2.8	3	3	3.2	3.2
**3**	man	oral	TDL	4.17	4	3.9	4	4	4	4.2	4.4
**4**	mouse	intraperitoneal	LD_{50}	2.9	4	4	3.3	3.4	3.4	3.6	3.6
**5**	mouse	intraperitoneal	LDL	2.97	4	4.1	3.4	3.5	3.5	3.7	3.7
**6**	mouse	intraperitoneal	TDL	4.29	4.7	4.7	4.5	4.6	4.5	4.7	4.8
**7**	mouse	intravenous	LD_{50}	3.45	4.4	4.5	4	4.1	4	4.2	4.2
**8**	mouse	oral	LD_{50}	2.4	3.1	3.1	2.4	2.6	2.5	2.7	2.8
**9**	mouse	oral	LDL	2.51	3.1	3.1	2.5	2.7	2.6	2.8	2.9
**10**	mouse	oral	TDL	4.2	4.1	4.2	4.1	4.1	4.1	4.2	4.3
**11**	mouse	subcutaneous	LD_{50}	2.64	3.7	3.8	3	3.1	3.1	3.3	3.3
**12**	mouse	subcutaneous	LDL	2.79	3.6	3.6	3	3.1	3	3.3	3.3
**13**	mouse	unreported	LD_{50}	2.73	3.8	3.8	3.1	3.2	3.1	3.4	3.4
**14**	rat	intraperitoneal	LD_{50}	2.91	4	4.1	3.3	3.5	3.4	3.6	3.6
**15**	rat	intraperitoneal	LDL	3.01	3.9	3.9	3.3	3.4	3.4	3.5	3.5
**16**	rat	intraperitoneal	TDL	4.4	4.7	4.7	4.6	4.7	4.7	4.8	4.9
**17**	rat	intravenous	LD_{50}	3.41	4.5	4.6	3.9	4.1	4	4.3	4.3
**18**	rat	intravenous	TDL	5.12	5.5	5.5	5.6	5.7	5.6	5.8	5.9
**19**	rat	oral	LD_{50}	2.34	3	3.1	2.3	2.4	2.4	2.6	2.6
**20**	rat	oral	LDL	2.56	3	3.1	2.4	2.6	2.5	2.7	2.8
**21**	rat	oral	TDL	4.03	4.2	4.2	3.9	4	4	4.1	4.3
**22**	rat	subcutaneous	LD_{50}	2.54	3.5	3.6	2.7	2.9	2.9	3	3
**23**	rat	subcutaneous	TDL	4.75	4.8	4.9	4.8	4.8	4.8	4.9	5.1
**24**	rat	skin	LD_{50}	2.03	2.3	2.4	1.8	1.9	2	2	2.1
**25**	rabbit	unreported	LD_{50}	2.69	3.5	3.5	2.8	3	2.9	3.1	3.2
**26**	rabbit	intravenous	LD_{50}	3.7	4.7	4.7	4.2	4.3	4.2	4.5	4.5

**27**	rabbit	oral	LD_{50}	2.43	3.1	3.1	2.4	2.6	2.5	2.8	2.8
**28**	rabbit	skin	LD_{50}	2.06	2.8	2.9	2.2	2.4	2.4	2.5	2.5
**29**	woman	skin	TDL	4.06	3.9	3.9	3.9	3.9	3.9	4	4.2

LD_{50}- Lethal Dose Fifty; TDL -Toxic Dose Low; LDL- Lethal Dose Low.

Table S6. The toxicity predictions of oral toxicity (LD_{50}, mg/kg) of tested ILs to different animal species (values were converted to mg/kg from Table S5)

ILs	Guinea pig	Mouse	Rat	Rabbit
3	188	236	297	247
4	215	258	310	258
13	907	1370	1900	1250
16	577	873	1200	834
17	852	1120	1620	1120
20	365	552	728	504
22	317	459	633	418

Table S7. The prediction of dermal toxicity (LD_{50}, mg/kg) of tested ILs to different animal species (values were converted to mg/kg from Table S5)

ILs	Rat	Rabbit
3	1420	410
4	1560	449
13	4990	2080
16	3640	1320
17	4270	1620
20	2900	917
22	2300	834

2. *Molecular docking of ligands as potential inhibitors of A.baumannii* and *S. aureus Enoyl-ACP reductase*

The alignments (Figure S3) indicated a significant enzymes similarity of AFabI and SFabI: 47% sequence identity, 64% sequence similarity and low number of gaps (1%). The enzyme secondary structures of AFabI (PDB ID: 6AH9) and the SFabI (PDB ID: 3GR6) were also compared using the UCSF Chimera program [3] (Figure S3).

Figure 3S presents a comparative analysis of the primary structure of FabI *A.baumannii* (AFabI) (UniProtKB: D0CAD5) [4] and FabI *S. aureus* (SFabI) (UniProtKB: Q6GI75) [5] using NCBI Protein BLAST [6].

```
Score           Expect  Method                         Identities      Positives       Gaps
224 bits(571)   1e-77   Compositional matrix adjust.   120/254(47%)    164/254(64%)    4/254(1%)

Query  27    LAGKRFLIAGVASKLSIAYGIAQALHREGAELAFTYPNEKLKKRVDEFAEQFGSK--LVF  84
             L  K ++I G+A+K SIA+G+A+ L + GA+L FTY  E+ +K +++  EQ       ++
Sbjct  4     LENKTYVIMGIANKRSIAFGVAKVLDQLGAKLVFTYRKERSRKELEKLLEQLNQPEAHLY  63

Query  85    PCDVAVDAEIDNAFAELAKHWDGVDGVVHSIGFAPAHTLDGDFTDVTDRDGFKIAHDISA  144
               DV  D E+ N F ++ K    +DGV HSI FA    L G F++ T R+GF +A DIS+
Sbjct  64    QIDVQSDEEVINGFEQIGKDVGNIDGVYHSIAFANMEDLRGRFSE-TSREGFLLAQDISS  122

Query  145   YSFVAMARAAKPLLQARQGCLLTLTYQGSERVMPNYNVMGMAKASLEAGVRYLASSLGVD  204
             YS   +A  AK L+    G ++  TY G E  + NYNVMG+AKASLEA V+YLA  LG D
Sbjct  123   YSLTIVAHEAKKLM-PEGGSIVATTYLGGEFAVQNYNVMGVAKASLEANVKYLALDLGPD  181

Query  205   GIRVNAISAGPIRTLAASGIKSFRKMLDANEKVAPLKRNVTIEEVGNAALFLCSPWASGI  264
              IRVNAISAGPIRTL+A G+  F  +L   E+ APLKRNV   EVG  A +L S  +SG+
Sbjct  182   NIRVNAISAGPIRTLSAKGVGGFNTILKEIEERAPLKRNVDQVEVGKTAAYLLSDLSSGV  241

Query  265   TGEILYVDAGFNTV  278
             TGE ++VD+GF+ +
Sbjct  242   TGENIHVDSGFHAI  255
```

Figure S3. Protein BLAST results of AFabI (278 amino acid residues) and SFabI (255 amino acid residues).

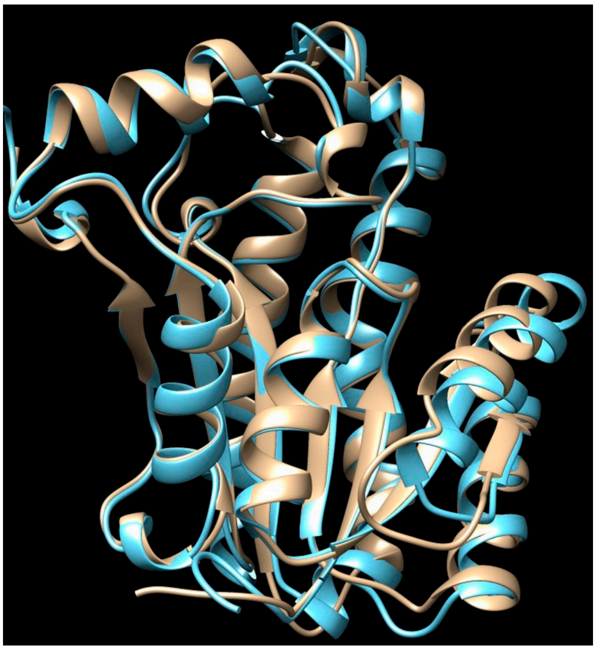

Figure S4. The enzyme secondary structures of AFabI (PDB ID: 6AH9) (blue) and SFabI (PDB ID: 3GR6) (beige).

Figure S4 also shows a high similarity of the secondary structures of studied enzymes. The triclosan-binding region AFabI and SFabI were used in the docking procedure based on the structural analysis data of 6AH9 and 3GR6.

Ligands **3**, **4**, and **16**, as the most active against both microbial pathogens, were docked into the triclosan-binding region of AFabI and SFabI (Figure S5-S7 and Table S8, S9).

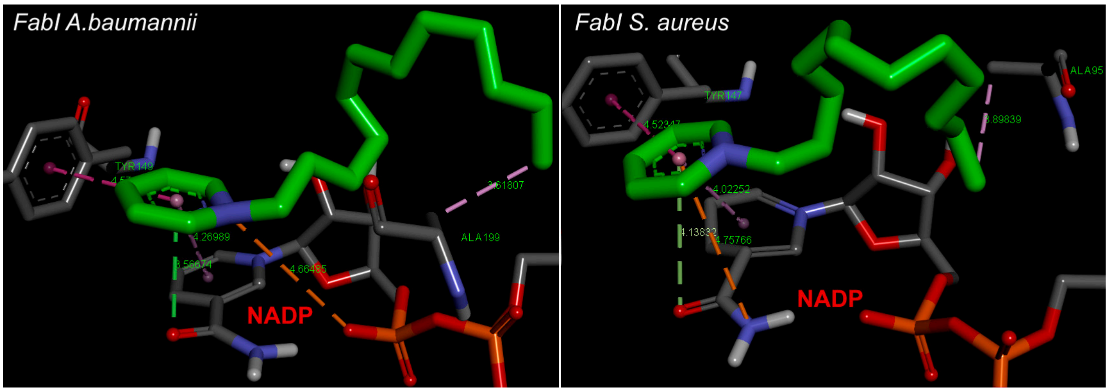

Figure S5. Molecular docking of the ligand **3** into the active site of AFabI and SFabI.

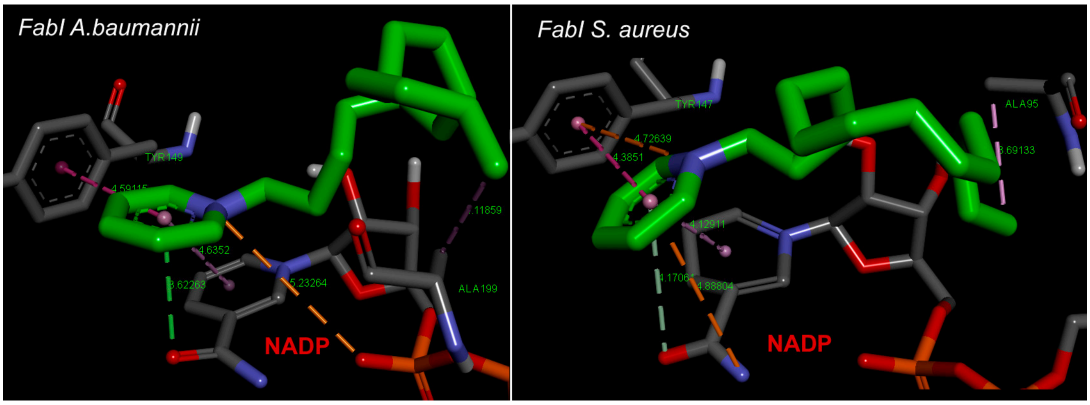

Figure S6. Molecular docking of the ligand **4** into the active site of AFabI and SFabI.

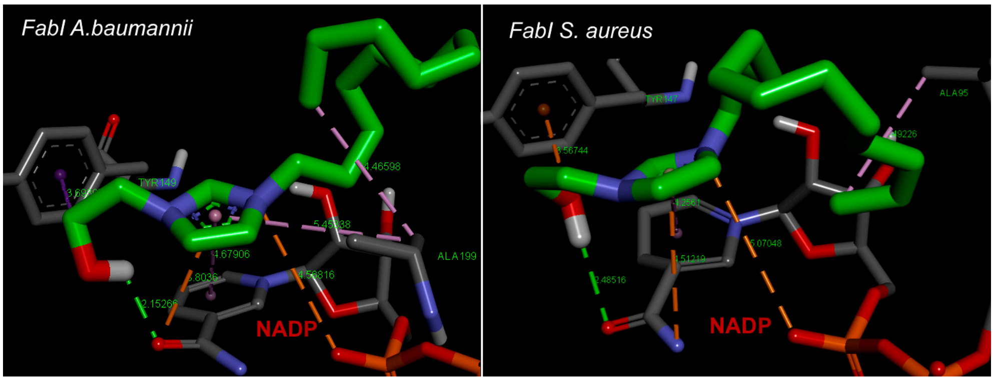

Figure S7. Molecular docking of the ligand **16** into the active site of AFabI and SFabI.

Table S8. Docking results of ligands **3**, **4, 16** into AFabI active sites.

Compound	Docking Score, (kcal/mol)	*Ki*, (uM)*	Hydrogen bonds	Electrostatic interaction	Hydrophobic interactions
**3**	−6.72	12.31	NADP (3.56Å)	NADP (4.66Å)	NADP (4.27Å), TYR149 (4.53Å), ALA199 (3.61Å)
**4**	−6.70	11.92	NADP (3.62Å)	NADP (5.23 Å)	NADP (4.63Å), TYR149 (4.59Å), ALA199 (4.19Å)
**16**	−6.50	13.6	NADP (2.15Å)	NADP (3.80Å), NADP (4.57Å)	NADP (4.67Å), TYR149 (3.69Å), ALA199 (4.46Å), ALA199 (5.45Å)

*The K_i values were estimated based on the dockings scores.

Table S9. Docking results of ligands **3**, **4, 16** into SFabI active sites.

Compound	Docking Score, (kcal/mol)	Ki, (uM)*	Hydrogen bonds	Electrostatic interaction	Hydrophobic interactions
**3**	−6.93	8.3	NADP (4.13Å)	NADP (4.75Å)	NADP (4.02Å), TYR147 (4.52Å), ALA95 (3.89Å)
**4**	−6.71	12.1	NADP (4.17Å)	NADP (4.88Å)	NADP (4.13Å), TYR147(4.38Å), TYR147(4.72Å), ALA95 (3.69Å)
**16**	−6.60	12.7	NADP (2.48Å)	NADP (4.51Å), NADP (5.07Å), TYR147(3.56Å)	NADP (4.67Å), NADP (4.25Å), ALA95 (4.49Å)

*The K_i values were estimated based on the dockings scores.

Thus (Table S8, S9) the formation of ligand-protein complexes is accompanied by estimated binding energies in the similar ranges from − 6.5 to − 6.72 kcal/mol (AFabI) and from − 6.6 to − 6.93 kcal/mol (SFabI). The measured *in silico* enzymes inhibition constant (Ki) as a binding affinity of ligands 3, 4, 16 for AFabI and SFabI target enzymes, are the values equal order (8.33 − 13.55 uM). The ligand-protein complexes were stabilized through hydrogen bonds (2.15 – 4.17Å), electrostatic (3.56 – 5.23Å) and hydrophobic (3.61– 5.45Å) interactions. The key role in complexation belongs to ALA95, TYR147 (TYR149), ALA199 amino acids and cofactor NADP.

References

1. Sushko, I.; Novotarskyi, S.; Korner, R.; Pandey, A.K.; Rupp, M.; Teetz, W.; Brandmaier, S.; Abdelaziz, A.; Prokopenko, V.V.; Tanchuk, V.Y., et al. Online chemical modeling environment (OCHEM): web platform for data storage, model development and publishing of chemical information. *J. Comput. Aided. Mol. Des.* **2011**, *25*, 533-554, doi:10.1007/s10822-011-9440-2.
2. Sosnin, S.; Karlov, D.; Tetko, I.V.; Fedorov, M.V. Comparative Study of Multitask Toxicity Modeling on a Broad Chemical Space. *J. Chem. Inf. Model.* **2019**, *59*, 1062-1072, doi:10.1021/acs.jcim.8b00685.

3. Pettersen, E.F.; Goddard, T.D.; Huang, C.C.; Couch, G.S.; Greenblatt, D.M.; Meng, E.C.; Ferrin, T.E. UCSF Chimera—A visualization system for exploratory research and analysis. *J. Comp. Chem.* **2004**, *25*, 1605-1612, doi:10.1002/jcc.20084.
4. UniProtKB. Enoyl-[acyl-carrier-protein] reductase [NADH]. Availabe online: https://www.uniprot.org/uniprot/D0CAD5 (accessed on 25 December 2020).
5. Enoyl-[acyl-carrier-protein] reductase [NADPH] FabI. Availabe online: https://www.uniprot.org/uniprot/Q6GI75 (accessed on 25 December 2020).
6. Altschul, S.F.; Madden, T.L.; Schaffer, A.A.; Zhang, J.; Zhang, Z.; Miller, W.; Lipman, D.J. Gapped BLAST and PSI-BLAST: a new generation of protein database search programs. *Nucleic Acids Res.* **1997**, *25*, 3389-3402., doi:10.1093/nar/25.17.3389.
